# Re-Emergence and Characterization of a Highly Pathogenic Getah Virus on a Pig Farm in Guangdong Province, China

**DOI:** 10.3390/microorganisms14040846

**Published:** 2026-04-09

**Authors:** Handuo Jia, Huahua Kang, Pinpin Chu, Tongqi Wang, Yulin Guo, Jitong Chen, Jiaxi Li, Xia Zhou, Duo-Liang Ran, Li-Yin Du, Shao-Lun Zhai

**Affiliations:** 1 College of Veterinary Medicine, Xinjiang Agricultural University, Urumqi 830052, China; jiahanduo@163.com (H.J.); 18928441819@163.com (T.W.); xjrdl7@163.com (D.-L.R.); 2 Guangdong Provincial Key Laboratory of Livestock Disease Prevention, Scientific Observation and Experiment Station of Veterinary Drugs and Diagnostic Techniques of Guangdong Province, Institute of Animal Health, Guangdong Academy of Agricultural Sciences, Guangzhou 510640, China; huahuakang2005@163.com (H.K.); cpp1900@163.com (P.C.); yulinguo1022@163.com (Y.G.); 15019311017@163.com (J.C.); jassie_lkh0505@163.com (J.L.); xiazhou698868@163.com (X.Z.); 3 Zijin Animal Disease Prevention and Control Center, Zijin 517400, China

**Keywords:** getah virus, re-emergence, sequence analysis, genetic diversity, pathogenicity

## Abstract

Getah virus (GETV), a mosquito-borne virus capable of infecting multiple economically important animal species, poses a potential epidemic risk. In May 2024, one pig farm from Heyuan, Guangdong Province, China, suffered reproductive disorders in sows and diarrhea in newborn piglets. Out of the six blood samples that were collected, three tested strongly positive for GETV, yielding a positivity rate of 50%. Moreover, a GETV strain (designated GDHYLC2024) was successfully isolated and identified. The viral titer of GDHYLC2024 was 10^7.687^ TCID_50_/mL in Vero cells. Its genome was composed of 11,688 bases in length. Interestingly, compared with GDHYLC23, it had no unique 32-nucleotide repeat insertion in 3′ non-coding region. However, phylogenetic analysis showed that GDHYLC2024 and GDHYLC23 clustered in genotype III. Animal infection experiments demonstrated that the GDHYLC2024 strain was highly pathogenic to 4-day-old piglets, which caused obvious clinical symptoms including fever, depression, anorexia, periorbital edema, ataxia, and three deaths out of a total of five individuals in the infection group. This study reported re-emergence of GETV in the same region of Guangdong Province, China. The above findings suggest that GETV continuously poses a threat to farm pig’s health and has genetic diversity.

## 1. Introduction

Getah virus (GETV), an RNA virus, belongs to the Alphavirus genus of the Togaviridae family [1]. Alphaviruses are arthropod-transmitted pathogens, so GETV is carried and transmitted by mosquitoes including the species of *Armigeres subalbatus*, *Anopheles sinensis*, *Culex annulus*, *Culex fuscocephala*, *Culex pseudovishnui*, and *Culex tritaeniorhynchus* [2]. Mosquito-borne arboviruses pose a serious threat to public health, as their vectors, mosquitoes, are widely distributed and frequently move across the globe, potentially leading to viral mutations with enhanced transmissibility [3].

GETV was firstly isolated from Culex gelidus mosquitoes in Malaysia in 1955. Subsequently, it has been detected in at least twelve countries including China, South Korea, Japan, Russia, Thailand, Mongolia, India, Australia, the Philippines, Sri Lanka, Cambodia, and Vietnam [4,5,6,7,8,9,10]. GETV is spreading polewardly from tropical to temperate regions. Notably, GETV has been reported even in areas with harsh climatic conditions [11]. The initial isolates were all from mosquitoes and no GETV infection had been reported in other animals. Until 1978, the first outbreak with GETV infection was identified in horses with fever, rash, and edema of the limbs in a training stable in Japan. Six years later, in 1985, another GETV outbreak was reported in Japanese swine herd [12,13]. Since then, its distribution and host range have gradually expanded. Other hosts such as cattle [14], wild boars [15], blue foxes [16], and red pandas [17] infected with GETV were reported. Under experimental conditions, GETV can also infect several laboratory animals including mice, hamsters, rabbits, apes, orangutans, and guinea pigs. In addition, serological surveys have revealed the presence of antibodies to GETV in other animals such as sheep, goats, kangaroos, monkeys, dogs, chicken, duck, and some wild birds, and even humans [4,18]. With the continuous expansion of its host range, GETV should be closely monitored.

Pigs are the main natural host of GETV, among which pregnant sows and newborn piglets are more susceptible to infection. Piglets infected by GETV show diarrhea, neurological symptoms, depression, and even death, and infected sows manifest reproductive failure including abortions, miscarriage, and stillbirths [19]. There has been an increasing number of reports on GETV infections in pigs. Significantly, in 2023, a novel GETV strain (designated as GDHYLC2023) with repetitive sequence insertion in 3′ non-coding region was isolated, which caused reproductive disorder in sixty-two percent of sows (310 of 500) and death in 80% of newborn piglets (284 of 356) from one pig farm in Heyuan city, Guangdong Province, China [20]. One year later, another swine farm in the same city also experienced GETV infection. In this study, a different GETV strain (designated as GDHYLC2024) with GDHYLC2023 was isolated from the infection case. Moreover, its genome characterization and pathogenicity were further identified.

## 2. Materials and Methods

### 2.1. Sample Collection and Detection

In early May 2024, an outbreak of reproductive disorders characterized by abortion, stillbirth, and rapid death of newborn piglets was observed on a pig farm in Heyuan City, Guangdong Province, China. Six blood samples collected from affected sows were submitted for testing and stored at −80 °C for further analysis. Viral nucleic acid was extracted using a MagPure Viral DNA/RNA Mini LQ Kit (Magen, Guangzhou, China). The samples were tested by one-step quantitative RT-PCR (qRT-PCR) for common swine pathogens associated with reproductive disorders and neonatal mortality, including respiratory syndrome virus (PRRSV), porcine rotavirus (PoRV), porcine epidemic diarrhea virus (PEDV), transmissible gastroenteritis virus (TGEV), and porcine deltacoronavirus (PDCoV). Subsequently, the samples were tested for GETV RNA by qRT-PCR using the PrimeScript One Step RT-PCR Kit Ver. 2 (Takara, Dalian, China), with primers and probe sequences described in the previous literature [21].

### 2.2. Virus Isolation and Identification

Positive serum samples were filtered through 0.22 µm filter and then inoculated on Vero cells. After 1 h of incubation, the supernatant was meticulously removed. The cells were subsequently incubated in a 5% CO_2_ incubator until the cytopathic effect (CPE) was observed or continuously blind-passaged for three generations. The obtained GETV isolate was designated as strain GDHYLC2024 following qRT-PCR detection of different passages of the isolated virus.

### 2.3. Viral Purification and Growth Kinetics

The GETV isolate was purified by plaque cloning in Vero cells. Virus supernatant was serially diluted and inoculated onto confluent monolayers of Vero cells in 12-well plates, followed by incubation at 37 °C with 5% CO_2_ for 90 min. Subsequently, the medium was removed, and cells in each well were overlaid with 2 mL of a 1:1 mixture of 2 × Dulbecco’s Modified Eagle Medium (DMEM) and 1.8% agarose. After the overlay solidified, cells were cultured at 37 °C under 5% CO_2_ for 2–5 days to allow for plaque development. Once plaques reached an appropriate size, neutral red staining was performed for 25 min to visualize plaque morphology. Individual, well-isolated plaques were picked with a pipette tip, dissolved in 300 µL of DMEM, and subjected to three freeze–thaw cycles. The resulting plaque suspension was then inoculated onto fresh Vero cells for amplification in preparation for the next round of plaque purification. The plaque purification procedure was repeated three times in total. The titer of the purified virus was determined using Vero cells. GETV viral solution was serially diluted in 10-fold steps from 10^−1^ to 10^−10^ using DMEM maintenance medium, and 100 µL of each dilution was inoculated into confluent monolayers of Vero cells in 96-well plates. The assay was performed in triplicate, with eight parallel wells per dilution. The plates were then incubated continuously at 37 °C under 5% CO_2_ for 5 days, with daily observation and recording of cytopathic effects, and the Reed–Muench method was used to determine the tissue culture infectious dose 50 (TCID_50_).

A viral growth curve of the GDHYLC2024 strain in Vero cells was constructed based on TCID_50_. Cells in 6-well plates were infected with different multiplicities of infection (MOI = 0.01, 0.1, and 1) and incubated at 37 °C for 1 h. After adsorption, the Vero cells were washed twice with PBS and maintained in 2 mL of DMEM. Whole-well lysates were collected at 6, 12, 24, 36, 48, 60, and 72 h post-infection (hpi), subjected to two freeze–thaw cycles at −80 °C and room temperature, and then viral titers were determined by the TCID_50_ using the Reed–Muench method.

### 2.4. Complete Genome Sequencing and Analyses

Nucleic acid was extracted from the third-passage virus culture supernatant; subsequently, the full genome was amplified using 13 pairs of primers described in a previous study [2]. The fragments were amplified by PCR using an amplification kit (TaKaRa, Dalian, China). Amplification products were cloned into the pMD19-T simple vector (Takara, Dalian, China) and then sequenced (Sangon Biotech, Guangzhou, China). The sequences were analyzed and assembled using SnapGene Viewer (version 6.0.2). The genome obtained was subjected to identity search with BLAST (https://blast.ncbi.nlm.nih.gov/Blast.cgi, accessed on 19 May 2025) and was aligned with reference sequences from the GenBank database (https://www.ncbi.nlm.nih.gov/genbank/, accessed on 19 May 2025) using BioEdit (version 7.2.5.0). A maximum likelihood phylogenetic tree was constructed using MEGA 11 with 1000 bootstrap replicates and rooted at the midpoint. The phylogenetic tree was visualized and refined using iTOL (https://itol.embl.de, accessed on 19 May 2025), and sequence identity analysis along with amino acid sequence alignment were performed using DNASTAR Lasergene software suite (version 17, DNASTAR Inc., Madison, WI, USA). Amino acid sequence alignments were generated using ESPript 3.0 (https://espript.ibcp.fr/ESPript/cgi-bin/ESPript.cgi, accessed on 19 May 2025).

### 2.5. Pathogenicity Study

To evaluate the pathogenicity of the GDHYLC2024 strain on piglets, we screened and selected 10 four-day-old piglets that tested negative for classical swine fever virus (CSFV), porcine reproductive and PRRSV, pseudorabies virus (PRV), PEDV, TGEV, PDCoV, PoRV, and GETV antigens, as well as for GETV antibodies. The 10 four-day-old piglets were randomly divided into two groups: one infected group and another control group (5 piglets per group). Each group was housed in separate isolation rooms with constant access to food and water. The pigs in the infected group received an intramuscular injection of 2 mL of GDHYLC2024 (1 × 10^6^ TCID_50_/mL), whereas those in the control group received an intramuscular injection of 2 mL of DMEM. Daily measurements of body temperature, weight, and clinical symptoms were recorded for each group. Oral swabs, fecal swabs, and blood samples were collected at 0, 6, 24, 48, 72, 96, and 120 hpi for viral load detection via RT-qPCR. Throughout the experiment, piglet survival was recorded daily. Any piglets that died before 120 hpi were necropsied immediately. At 120 hpi, all surviving piglets were euthanized and necropsied. Spleen, lung, kidney, cerebellum, jejunum, inguinal lymph nodes, and testes were collected from each piglet.

### 2.6. Statistical Analysis

All statistical analyses were performed using GraphPad Prism software (version 10.1.2). Clinical and virological data (viral load, body temperature) are presented as mean ± SD. To assess statistically significant differences among the experimental groups, a two-way analysis of variance (ANOVA) was conducted. The threshold for statistical significance was set at *p* < 0.05.

### 2.7. Ethical Approval

All of the experiments were performed at the Laboratory Animal Care of the Institute of Animal Health, Guangdong Academy of Agricultural Sciences. The experiments (No. YC-PT2025006) were approved and conducted per the relevant guidelines with strict biosecurity measures and hygiene procedures.

## 3. Results

### 3.1. Pathogen Detection in Blood Samples

All blood samples were tested by qRT-PCR and were negative for PRRSV, PoRV, PEDV, TGEV, and PDCoV, whereas GETV RNA was detected in 3 out of 6 samples (50%), with Ct values of 18.152, 17.434, and 16.215.

### 3.2. Virus Isolation, Purification, and Growth Kinetics

**Figure 1:**
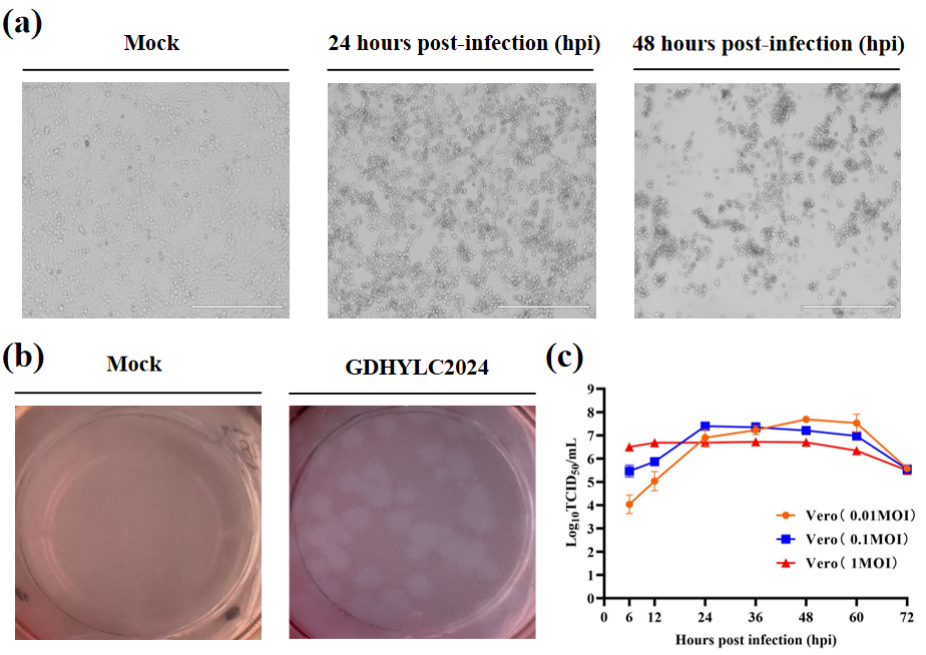
Isolation, purification, and growth kinetics of the GETV strain of GDHYLC2024. (**a**) CPE observed in Vero cells at 24 and 48 hpi with the GDHYLC2024 strain, scale bar: 400 μm. (**b**) Plaques formed following inoculation with a 10^4^ dilution of the virus stock. (**c**) Growth kinetics of GDHYLC2024 in Vero Cells. Infection with GETV at 0.01, 0.1, and 1 MOIs. All values are presented as mean ± SD from three independent experiments.

The three samples that tested positive for GETV were filtered and inoculated onto Vero cells for virus isolation. CPE was observed in one of the samples after a single passage, characterized by cell aggregation, shrinkage, and detachment as early as 20 hpi (Figure 1a). The supernatant was harvested, and subsequent RT-PCR amplification and sequencing confirmed the presence of GETV, which was designated as GDHYLC2024. Following three rounds of plaque purification, the plaques appeared uniform with well-defined edges (Figure 1b). The titer of the purified virus stock after three passages was determined to be 10^6.8^ TCID_50_/mL, as calculated by the Reed–Muench method. A growth curve was subsequently generated by infected Vero cells at different MOI (Figure 1c). Consequently, the highest viral titer, reaching 10^7.687^ TCID_50_/mL, was achieved at 48 hpi with 0.01 MOI.

### 3.3. Genome Characterization and Phylogenetic Analysis

The genome of GDHYLC2024 was obtained through de novo assembly of sequenced fragment and was submitted to GenBank database (accession number: PQ662933). Viral genome was 11,688 nucleotides (nts) in length with a GC content of 52.23% (excluding the poly-A tail), composed of four regions including a 5′-untranslated region (5-UTR), two open reading frames (ORFs), and a 3′-UTR. The first ORF contained 7404 nts encoding a polyprotein that was processed four non-structural proteins (nsp1-nsp4), followed by the second ORF, which contained 3762 nts encoding the structural protein precursor (C-6K-E2-E3-E1).

A total of 40 complete genomes, including several representative strains such as the prototype strain MM2021, the Hunan isolate HuN1, and the first Hainan isolate M1, were retrieved from the GenBank database. Sequence identity analysis revealed (Table 1) that the whole genome of strain GDHYLC2024 exhibited the highest nucleotide identity (99.6%) with GETV-AH. Compared with the GDHYLC23 strain isolated from a diseased pig in Heyuan, Guangdong Province, in 2023, it also exhibited a high nucleotide identity (98.6%). In contrast, it exhibited the lowest nucleotide identity (95.2%) with GETV-China/GX2020, a GIV genotype strain isolated from one pangolin in Guangxi, China. Moreover, the sequence identity of the structural and non-structural genes of the GDHYLC2024 strain showed a trend similar to that of the whole-genome analysis. Notably, the capsid (Cap) protein was more conserved, and reference strains generally exhibited higher identity with the GDHYLC2024 strain in this region. The E2 gene identity of this strain was highest with GETV-AH (99.8%) and was also highly identical to the Chinese isolate GETV-GDFS2-2018 from 2018 (99.5%). Importantly, comparative analysis of the E2 amino acid sequences identified a unique mutation (I367V at residue 367) in the GDHYLC2024 strain (Figure S1).

**Figure 2:**
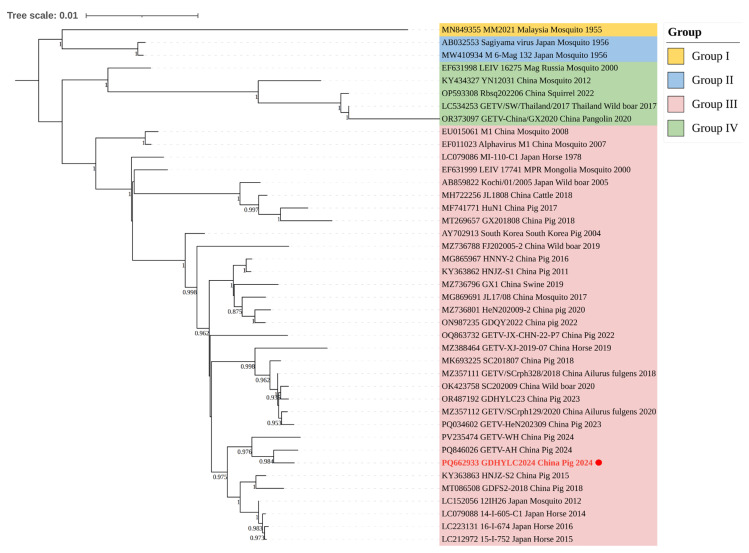
Maximum likelihood phylogenetic tree based on the complete genome sequences of GETV. Bootstrap values (≥0.70) are shown at the nodes. The scale bar indicates nucleotide substitutions per site. The strain GDHYLC2024 isolated in this study is indicated with a red dot.

For subsequent alignment and phylogenetic analysis, phylogenetic trees were constructed based on the complete genome and the nucleotide sequences of the non-structural, structural, NSP1, NSP2, Cap, E2, and E1 genes using the maximum likelihood method in MEGA11 software, with 1000 bootstrap replicates. The tree based on the complete genome evolved into four distinct genetic groups (Figure 2). The remaining gene-specific trees are provided in the Supplementary Materials (Figures S2–S8). Phylogenetic trees constructed from different genomic regions consistently classified the GDHYLC2024 strain into Group III. These results indicated that this strain is closely related to the Chinese strains HNJZ-S2 and GDFS-2018 archived in GenBank. Further phylogenetic analysis revealed its closest relationship to the 2024 porcine GETV-AH strain from China.

### 3.4. Pathogenicity Analysis

**Figure 3:**
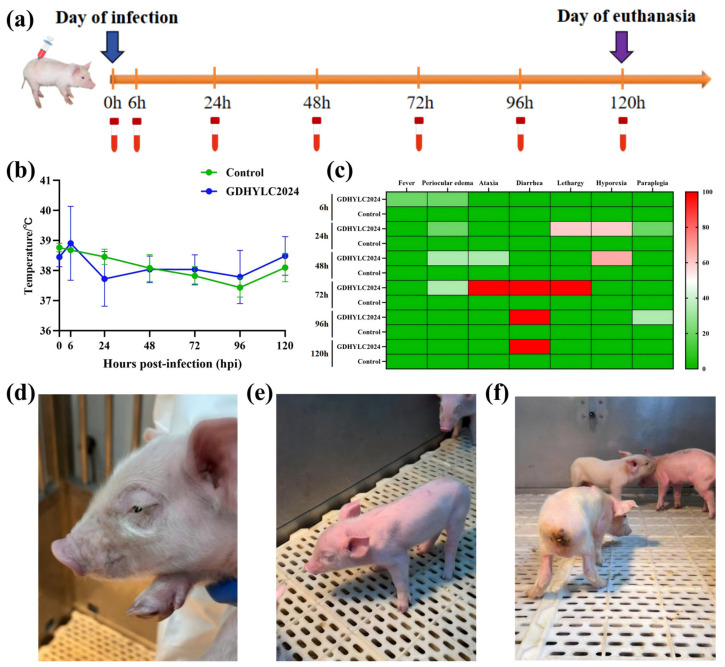
Experiment design and clinical presentations. (**a**) Experiment infection design timeline. (**b**) Monitoring of rectal temperature. Rectal temperature exceeding 40 °C was defined as fever. (**c**) Clinical symptom scoring. (**d**–**f**) Clinical symptoms induced by the infection of GDHYLC2024, including ocular edema, ataxia, and diarrhea.

To comprehensively assess the pathogenicity of the GDHYLC2024 strain, we conducted an infection experiment on 4-day-old piglets (Figure 3a) with temperature measurements, weight assessments, and collection of fecal and oral swabs along with blood samples performed at each time point. Pathogenicity analysis revealed that clinical symptoms in the infected piglets primarily included fever, ocular edema, ataxia, diarrhea, hindlimb paralysis, and death in severe cases. Within 6 hpi, one piglet in the infection group exhibited an elevated temperature reaching 41 °C (Figure 3b) accompanied by ocular edema. At 24 hpi, over half of the piglets displayed lethargy and reduced appetite. At 48 hpi, infected piglets developed diarrhea and ataxia, with two exhibiting severe paralysis (Figure 3c–f). Meanwhile, the control group maintained normal physiological body temperatures and good health throughout the experiment period.

**Figure 4:**
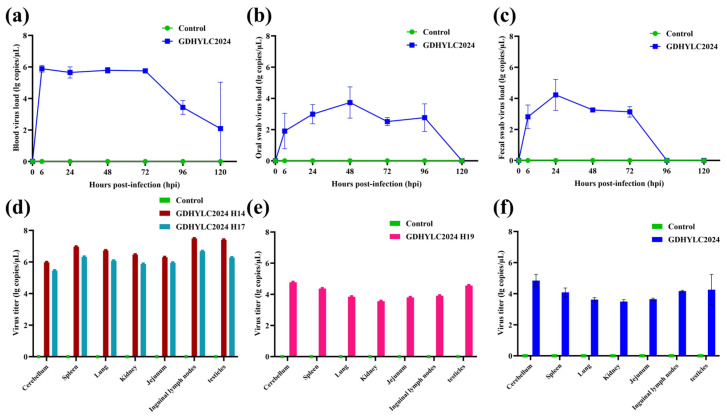
Viral load in tissues of piglets infected with GDHYLC2024, detected via viremia and viral shedding, as well as viral load in tissues of piglets that died during the experiment and at 120 h euthanasia. (**a**–**c**) Viral load in blood, oral swabs, and fecal swabs. (**d**) Viral load in different tissues during necropsy of 30 hpi dead piglets. (**e**) Viral load in different tissues during necropsy of piglets that died at 96 hpi. (**f**) At 120 hpi, pigs were euthanized, and viral loads in various tissues were quantified. Each bar represents the mean ± standard deviation for each group.

RT-qPCR was used to assess viral load and distribution in serum samples. Fecal swabs and oral swabs collected at 0, 6, 24, 48, 72, 96, and 120 hpi, as well as in seven tissues and organs (spleen, lung, kidney, cerebellum, jejunum, inguinal lymph nodes, and testes), were obtained during necropsy to determine GETV genome copy numbers for each piglet. Viremia levels in the infection group were initially detected at 6 hpi and reached high levels of replication. The peak viremia in the infection group occurred at 48 hpi, followed by a gradual decline in viral load. By 96 hpi, viremia levels in the infection group had significantly decreased (Figure 4a). Viral shedding monitoring revealed substantial viral presence in both oral and fecal swabs, with normality restored after 96 hpi or 120 hpi. No viral shedding or viremia was detected in the control group throughout the study period (Figure 4b,c). During the experiment, three piglets died in the infection group: two (nos. H14 and H17) at 30 hpi and one (no. H19) at 96 hpi. The detection results revealed high viral loads in the spleen, jejunum, and testes of two dead piglets (nos. H14 and H17), while the high viral loads appeared in the cerebellum, spleen, and testes of the dead piglet of H19 (Figure 4d,e). Therefore, during the early stage of infection, the viral load is primarily higher in the testes and inguinal lymph nodes; during the late stage of infection, while viral loads in peripheral tissues decline significantly, the cerebellum maintains relatively high viral titers.

**Figure 5:**
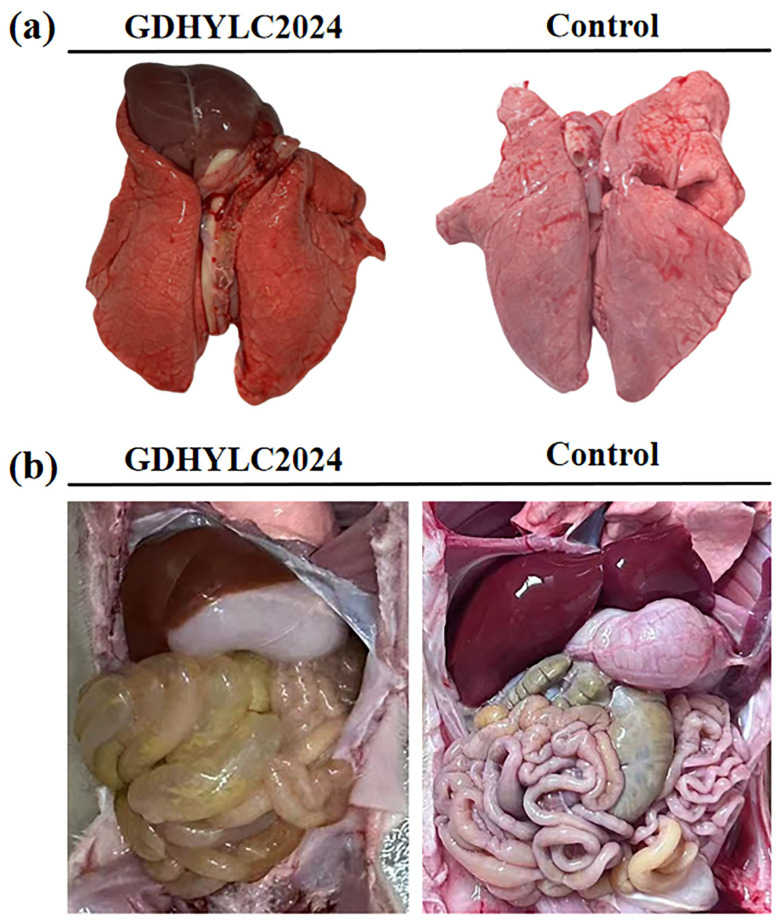
Necropsy findings. (**a**) Pulmonary hemorrhage. (**b**) Thinning of the small intestinal wall and jejunal pneumatosis.

In contrast, no piglets died in the control group throughout the experiment. Following euthanasia at 120 hpi, tissue samples were collected from all piglets. The results indicated that this strain caused multi-organ infection in piglets (Figure 4f), with high viral loads detected in the cerebellum, jejunum, and testes. Concurrently, necropsy of the challenged piglets revealed pulmonary hemorrhage and parenchymal hyperplasia, along with jejunal emphysema characterized by markedly thinned walls distended with yellowish serous fluid (Figure 5a,b).

This pathogenicity study demonstrated that the GDHYLC2024 strain rapidly induced disease onset in 4-day-old piglets. Within 6 hpi, some pigs exhibited elevated body temperature. All experimental infection pigs displayed anorexia and huddling behavior, with high viral genome copy numbers detectable in blood samples that persisted for 1–2 days before declining. Clinically, this strain causes eye edema and adhesion, diarrhea, ataxia, and hind limb paralysis, and severe cases may even result in death. Postmortem examination revealed that the virus causes multi-organ infection in piglets, with the most obvious gross lesions observed in the lungs and small intestine.

## 4. Discussion

As an emerging mosquito-borne virus, GETV is capable of infecting multiple animal species. Antibodies against GETV have been detected in human sera [22], posing a potential threat to global health security. In recent years, the detection rates of GETV antigens and antibodies have gradually increased across different provinces in China, particularly in swine herds, leading to significant economic losses. In 2024, intensive GETV outbreaks exhibiting epidemic characteristics occurred in pig farms across multiple regions of Henan Province, China [23]. GETV transmits through mosquito bites and is often overlooked. The virus can be spread by multiple mosquito species, making vector control an effective measure for its prevention and containment. Beyond mosquito-borne transmission, pigs can also be infected via the administration of contaminated vaccines. Two recent studies have detected GETV, including its novel strains, in commercially available PRRSV vaccines [24,25].

In this study, a GETV strain (GDHYLC2024) was successfully isolated and sequenced in 2024. The subsequent phylogenetic analysis encompassed a comprehensive set of classical GETV strains from across the globe. The results revealed that Group I consists solely of the prototype strain MM2021 isolated in 1955. Group II is composed of two strains isolated from mosquitoes in Japan. Group IV includes five strains isolated between 2000 and 2022 from diverse hosts, namely mosquitoes, squirrels, pangolins, and wild boars, showing a gradual increase in strain numbers within this group. However, to date, the third group remains the most widely distributed strain group. Amino acid sequence alignment of the E2 gene identified a unique mutation at position 367 (I367V) in the GDHYLC2024 strain. Recent studies have indicated that a single amino acid substitution at position 253 of the E2 protein can lead to attenuated virulence in GETV [26]. Furthermore, studies have shown that a single amino acid mutation in the GETV E2 protein can significantly regulate its virulence. Using reverse genetics, researchers introduced an N262D mutation (asparagine to aspartate at position 262) in the E2 protein of the epidemic strain SD2206. This point mutation disrupts an N-linked glycosylation site on the E2 protein, which in turn leads to markedly attenuated GETV virulence in animal models such as suckling mice and piglets [27]. Whether the I367V mutation influences viral pathogenicity warrants further investigation.

In recent years, pathogenicity studies have been conducted on some isolated GETV (GETV) strains. However, studies focusing specifically on their pathogenicity in 4-day-old piglets remain limited. As early as 1988, a comparative pathogenicity study was conducted in 4-week-old piglets and 8-month-old adult pigs. The results demonstrated that pigs of both age groups were susceptible to infection. The primary clinical manifestations included mild diarrhea and depression. Furthermore, transient viremia was detected in all challenge pigs, lasting for 1 to 2 days [19]. In recent years, as the number of isolated GETV strains in China has gradually increased, studies into its pathogenicity has correspondingly intensified. Previous studies have indicated that 10-day-old piglets infected with GETV developed clinical signs including fever, anorexia, and diarrhea, followed by ataxia and tremors, leading to death within one day. Postmortem examination revealed pathological manifestations such as interstitial pulmonary fibrosis, pulmonary hemorrhage, and splenomegaly with hemorrhage [28]. In 2024, one study team isolated a GETV strain from diarrheic pigs in Jiangxi Province and conducted a pathogenicity study in 10-day-old piglets. The results indicated that the infected piglets exhibited tremors, lethargy, and anorexia at 12 hpi, followed by mild diarrhea at 24 hpi. Postmortem examination revealed hemorrhages in the liver and spleen, pulmonary atrophy, and colonic edema [29]. In addition, pathogenicity studies have been conducted targeting 7-day-old piglets and pregnant sows. The results demonstrated that infected piglets developed diarrhea by 3 dpi, which was accompanied by fever. Postmortem examination of the piglets revealed thinning of the small intestinal wall, along with extensive hemorrhage and lesions in the lungs. In contrast, the infected pregnant sows did not exhibit obvious clinical signs. However, they still presented with a high level of viremia [30].

Multiple studies have demonstrated that GETV can infect 7- to 10-day-old piglets and induce high-level viremia. However, research on the pathogenicity of porcine-origin GETV isolates, specifically in 4-day-old piglets, remains scarce. In the present study, a pathogenicity investigation was conducted using 4-day-old piglets. The infected piglets exhibited exacerbated clinical signs, including ataxia, diarrhea, and periocular edema. More pronounced ataxia was observed at 48 hpi; this may be associated with the presence of substantial viral RNA loads in the cerebellum. However, the precise pathogenic mechanism underlying this association remains to be elucidated. Postmortem examination revealed pulmonary hemorrhage and lesions, as well as thinning of the small intestinal wall. Furthermore, persistent high viral loads were detected in the testes of infected boars. Given that GETV infection has been shown to reduce testosterone synthesis and impair sperm quality in mice [31], a critical question arises: could similar reproductive impairment occur in breeding boars, thereby posing a threat to swine breeding operations?

In summary, our study demonstrates that GETV GDHYLC2024 causes severe clinical signs and high mortality in 4-day-old piglets. However, it is important to note that the route of administration used in this study, intramuscular injection, differs from the natural route of GETV transmission via mosquito bites, which is intradermal or subcutaneous. This may have resulted in an overestimation of pathogenicity. Future studies should employ routes that more closely mimic natural infection to better evaluate virulence under field conditions.

In recent years, the number of GETV isolates identified in China has increased significantly, with outbreaks reported on pig farms across multiple provinces. This trend necessitates heightened attention and underscores the urgent need for implementing real-time surveillance and strengthened biosecurity measures.

## 5. Conclusions

In this study, we reported the re-emergence of GETV in Heyuan City, Guangdong Province, China. Furthermore, a novel strain of GDHYLC2024 was isolated, but genetically different from our previous reported GETV strain (GDHYLC2023) originating from the same region [20]. It indicated that GDHYLC2024 had a certain degree of genetic diversity. However, whether this genetic difference affects viral pathogenicity or epidemiological fitness remains to be determined. Nonetheless, pathogenicity experiments demonstrated that GDHYLC2024 was highly pathogenic for 4-day-old piglets. In recent years, during the backdrop of global warming, mosquito-borne diseases have emerged in localized outbreaks in some regions of China. As one of these mosquito-borne diseases, GETV has shown a sporadic epidemic trend in China. This study contributes to the epidemiology of GETV and informs subsequent diagnosis and vaccine research.

## Figures and Tables

**Table 1:** Sequence identity (%) analysis of GDHYLC2024 with other reference GETV strains.

GenBank No.	Strain	Complete Genome	Non-Structural Gene	Structural Gene	NSP1 Gene	NSP2 Gene	Cap Gene	E2 Gene	E1 Gene
PQ034602	GETV-HeN202309	98.6	98.7	98.5	98.8	98.7	98.8	98.7	98.4
OP593308	Rbsq202206	95.9	96.2	95.6	96.8	96.3	96.1	94.8	97.9
OR373097	GETV-China/GX2020	95.2	95.5	94.9	95.6	95.7	96.0	94.2	95.1
OR487192	GDHYLC23	98.6	98.8	98.6	98.7	98.8	98.6	98.7	98.6
AY702913	South Korea	99.0	99.0	99.1	99.1	99.0	99.3	98.9	99.2
LC152056	12IH26	99.2	99.2	99.4	98.9	99.1	99.1	99.4	99.8
LC079088	14-I-605-C1	99.2	99.1	99.3	98.9	99.1	99.1	99.3	99.6
LC212972	15-I-752	99.2	99.1	99.4	99.0	99.0	99.1	99.3	99.7
LC223131	16-I-674	99.2	99.1	99.3	98.9	99.1	99.1	99.3	99.6
EF011023	Alphavirus M1	98.0	98.0	97.9	98.1	98.2	97.9	98.0	97.9
MZ736788	FJ202005-2	97.9	98.7	98.3	98.4	98.9	98.6	98.2	98.1
ON987235	GDQY2022	98.5	98.8	99.0	98.8	98.6	99.0	99.1	99.2
MT086508	GDFS2-2018	99.0	99.0	99.2	98.8	98.9	98.8	99.5	99.5
OQ863732	GETV-JX-CHN-22-P7	98.7	98.7	98.9	98.6	98.7	98.8	99.1	99.0
MZ388464	GETV-XJ-2019-07	98.4	98.4	98.6	98.4	98.4	98.5	98.4	98.9
MZ357112	GETV/SCrph129/2020	98.4	98.7	98.6	98.8	98.8	98.8	98.8	98.5
MZ357111	GETV/SCrph328/2018	98.6	98.8	98.7	98.8	99.0	98.8	98.8	98.6
LC534253	GETV/SW/Thailand/2017	95.9	96.2	95.6	96.6	96.3	96.1	95.0	96.0
MZ736796	GX1	98.1	98.8	98.8	98.8	98.7	99.1	98.7	98.9
MT269657	GX201808	97.2	97.2	97.3	97.6	97.4	98.1	96.4	97.9
MZ736801	HeN202009-2	98.8	98.7	99.0	98.8	98.6	98.9	99.1	99.3
KY363862	HNJZ-S1	99.0	99.0	99.1	99.0	99.0	99.0	99.0	99.4
KY363863	HNJZ-S2	99.2	99.1	99.5	98.9	99.0	99.1	99.5	99.8
MG865967	HNNY-2	98.8	98.9	98.8	98.8	98.9	99.0	99.1	99.2
MF741771	HuN1	97.4	97.3	97.8	97.7	97.4	98.3	97.1	98.2
MG869691	JL17/08	98.9	98.9	99.0	98.6	99.0	99.0	99.1	99.0
MH722256	JL1808	97.6	97.7	97.6	97.9	97.6	98.0	97.1	98.1
AB859822	Kochi/01/2005	97.7	97.8	97.7	98.1	97.8	98.0	97.2	98.2
EF631998	LEIV 16275 Mag	97.5	97.6	97.4	97.8	97.6	97.3	97.8	97.4
EF631999	LEIV 17741 MPR	98.4	98.4	98.7	98.4	98.5	98.6	98.7	98.9
MW410934	M 6-Mag 132	97.2	97.3	97.1	97.4	97.7	97.0	97.2	97.2
EU015061	M1	97.9	98.0	97.8	97.6	98.2	97.5	97.9	98.0
LC079086	MI-110-C1	98.5	98.4	98.6	98.6	98.4	98.8	98.6	98.8
MN849355	MM2021	95.3	95.5	95.0	96.4	95.4	95.1	94.5	95.2
AB032553	Sagiyama virus	97.2	97.4	96.9	97.5	97.7	97.0	97.0	97.0
MK693225	SC201807	98.8	98.9	98.8	98.8	99.0	99.0	99.0	98.6
OK423758	SC202009	98.6	98.7	98.6	98.9	98.9	98.8	98.6	98.6
KY434327	YN12031	96.1	96.2	96.2	98.6	96.2	96.3	96.2	96.2
PV235474	GETV-WH	99.3	99.2	99.5	98.7	98.7	99.6	99.5	99.4
PQ846026	GETV-AH	99.6	99.6	99.7	99.4	99.4	99.9	99.8	99.6

## Data Availability

The data presented in this study are openly available in National Center for Biotechnology Information database at https://www.ncbi.nlm.nih.gov/nucleotide/, GDHYLC2024, accession number is PQ662933.

## Supplementary Materials

The following supporting information can be downloaded at https://www.mdpi.com/article/10.3390/microorganisms14040846/s1. Figure S1: Alignment of the amino acid sequences of the E2 gene. Figures S2–S8: Phylogenetic trees based on the non-structural, structural, NSP1, NSP2, Cap, E2, and E1 gene sequences of GETV.
