# Supplementary material for: Re-Emergence and Characterization of a Highly Pathogenic Getah Virus on a Pig Farm in Guangdong Province, China
Source: Microorganisms. 2026 Apr 9;14(4):846. doi: 10.3390/microorganisms14040846 (PMC13119205; doi:10.3390/microorganisms14040846)
Supplement: Supplementary file 1 [file microorganisms-14-00846-s001.zip › microorganisms-4221267-supplementary/Supplementary_Figures.zip/Figure S1.Alignment of the amino acid sequences of the E2 gene.pdf]

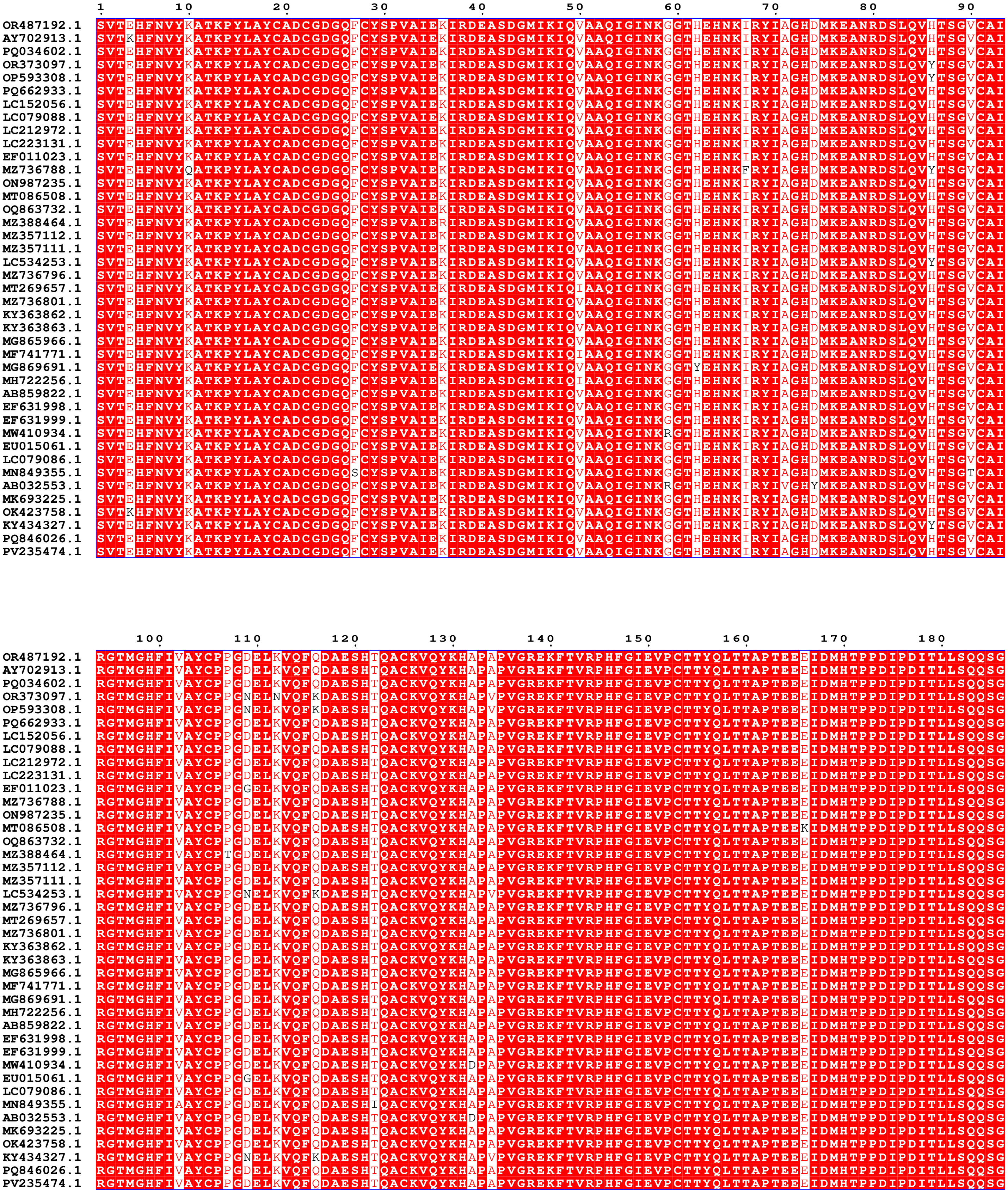
1 10 20 30 40 50 60 70 80 90
OR487192.1 SVTEHFNVYKATKPYLAYCADCGDGQFCYSPVAIEKIRDEASDGMIKIQVAAQIGINKGGTHEHNKIRYIAGHDMKEANRDSLQVHTSGVCAI
AY702913.1 SVTKHFNVYKATKPYLAYCADCGDGQFCYSPVAIEKIRDEASDGMIKIQVAAQIGINKGGTHEHNKIRYIAGHDMKEANRDSLQVHTSGVCAI
PQ034602.1 SVTEHFNVYKATKPYLAYCADCGDGQFCYSPVAIEKIRDEASDGMIKIQVAAQIGINKGGTHEHNKIRYIAGHDMKEANRDSLQVHTSGVCAI
OR373097.1 SVTEHFNVYKATKPYLAYCADCGDGQFCYSPVAIEKIRDEASDGMIKIQVAAQIGINKGGTHEHNKIRYIAGHDMKEANRDSLQVYTSGVCAI
OP593308.1 SVTEHFNVYKATKPYLAYCADCGDGQFCYSPVAIEKIRDEASDGMIKIQVAAQIGINKGGTHEHNKIRYIAGHDMKEANRDSLQVYTSGVCAI
PQ662933.1 SVTEHFNVYKATKPYLAYCADCGDGQFCYSPVAIEKIRDEASDGMIKIQVAAQIGINKGGTHEHNKIRYIAGHDMKEANRDSLQVHTSGVCAI
LC152056.1 SVTEHFNVYKATKPYLAYCADCGDGQFCYSPVAIEKIRDEASDGMIKIQVAAQIGINKGGTHEHNKIRYIAGHDMKEANRDSLQVHTSGVCAI
LC079088.1 SVTEHFNVYKATKPYLAYCADCGDGQFCYSPVAIEKIRDEASDGMIKIQVAAQIGINKGGTHEHNKIRYIAGHDMKEANRDSLQVHTSGVCAI
LC212972.1 SVTEHFNVYKATKPYLAYCADCGDGQFCYSPVAIEKIRDEASDGMIKIQVAAQIGINKGGTHEHNKIRYIAGHDMKEANRDSLQVHTSGVCAI
LC223131.1 SVTEHFNVYKATKPYLAYCADCGDGQFCYSPVAIEKIRDEASDGMIKIQVAAQIGINKGGTHEHNKIRYIAGHDMKEANRDSLQVHTSGVCAI
EF011023.1 SVTEHFNVYKATKPYLAYCADCGDGQFCYSPVAIEKIRDEASDGMIKIQVAAQIGINKGGTHEHNKIRYIAGHDMKEANRDSLQVHTSGVCAI
MZ736788.1 SVTEHFNVYQATKPYLAYCADCGDGQFCYSPVAIEKIRDEASDGMIKIQVAAQIGINKGGTHEHNKFRYIAGHDMKEANRDSLQVYTSGVCAI
ON987235.1 SVTEHFNVYKATKPYLAYCADCGDGQFCYSPVAIEKIRDEASDGMIKIQVAAQIGINKGGTHEHNKIRYIAGHDMKEANRDSLQVHTSGVCAI
MT086508.1 SVTEHFNVYKATKPYLAYCADCGDGQFCYSPVAIEKIRDEASDGMIKIQVAAQIGINKGGTHEHNKIRYIAGHDMKEANRDSLQVHTSGVCAI
OQ863732.1 SVTEHFNVYKATKPYLAYCADCGDGQFCYSPVAIEKIRDEASDGMIKIQVAAQIGINKGGTHEHNKIRYIAGHDMKEANRDSLQVHTSGVCAI
MZ388464.1 SVTEHFNVYKATKPYLAYCADCGDGQFCYSPVAIERIRDEASDGMIKIQVAAQIGINKGGTHEHNKIRYIAGHDMKEANRDSLQVHTSGVCAI
MZ357112.1 SVTEHFNVYKATKPYLAYCADCGDGQFCYSPVAIEKIRDEASDGMIKIQVAAQIGINKGGTHEHNKIRYIAGHDMKEANRDSLQVHTSGVCAI
MZ357111.1 SVTEHFNVYKATKPYLAYCADCGDGQFCYSPVAIEKIRDEASDGMIKIQVAAQIGINKGGTHEHNKIRYIAGHDMKEANRDSLQVHTSGVCAI
LC534253.1 SVTEHFNVYKATKPYLAYCADCGDGQFCYSPVAIEKIRDEASDGMIKIQVAAQIGINKGGTHEHNKIRYIAGHDMKEANRDSLQVYTSGVCAI
MZ736796.1 SVTEHFNVYKATKPYLAYCADCGDGQFCYSPVAIEKIRDEASDGMIKIQVAAQIGINKGGTHEHNKIRYIAGHDMKEANRDSLQVHTSGVCAI
MT269657.1 SVTEHFNVYKATKPYLAYCADCGDGQFCYSPVAIEKIRDEASDGMIKIQIAAQIGINKGGTHEHNKIRYIAGHDMKEANRDSLQVHTSGVCAI
MZ736801.1 SVTEHFNVYKATKPYLAYCADCGDGQFCYSPVAIEKIRDEASDGMIKIQVAAQIGINKGGTHEHNKIRYIAGHDMKEANRDSLQVHTSGVCAI
KY363862.1 SVTEHFNVYKATKPYLAYCADCGDGQFCYSPVAIEKIRDEASDGMIKIQVAAQIGINKGGTHEHNKIRYIAGHDMKEANRDSLQVHTSGVCAI
KY363863.1 SVTEHFNVYKATKPYLAYCADCGDGQFCYSPVAIEKIRDEASDGMIKIQVAAQIGINKGGTHEHNKIRYIAGHDMKEANRDSLQVHTSGVCAI
MG865966.1 SVTEHFNVYKATKPYLAYCADCGDGQFCYSPVAIEKIRDEASDGMIKIQVAAQIGINKGGTHEHNKIRYIAGHDMKEANRDSLQVHTSGVCAI
MF741771.1 SVTEHFNVYKATKPYLAYCADCGDGQFCYSPVAIEKIRDEASDGMIKIQIAAQIGINKGGTHEHNKIRYIAGHDMKEANRDSLQVHTSGVCAI
MG869691.1 SVTEHFNVYKATKPYLAYCADCGDGQFCYSPVAIEKIRDEASDGMIKIQVAAQIGINKGGTYEHNKIRYIAGHDMKEANRDSLQVHTSGVCAI
MH722256.1 SVTEHFNVYKATKPYLAYCADCGDGQFCYSPVAIEKIRDEASDGMIKIQIAAQIGINKGGTHEHNKIRYIAGHDMKEANRDSLQVHTSGVCAI
AB859822.1 SVTEHFNVYKATKPYLAYCADCGDGQFCYSPVAIEKIRDEASDGMIKIQVAAQIGINKGGTHEHNKIRYIAGHDMKEANRDSLQVHTSGVCAI
EF631998.1 SVTEHFNVYKATKPYLAYCADCGDGQFCYSPVAIEKIRDEASDGMIKIQVAAQIGINKGGTHEHNKIRYIAGHDMKEANRDSLQVHTSGVCAI
EF631999.1 SVTEHFNVYKATKPYLAYCADCGDGQFCYSPVAIEKIRDEASDGMIKIQVAAQIGINKGGTHEHNKIRYIAGHDMKEANRDSLQVHTSGVCAI
MW410934.1 SVTEHFNVYKATKPYLAYCADCGDGQFCYSPVAIEKIRDEASDGMIKIQVAAQIGINKRGTHEHNKIRYIAGHDMKEANRDSLQVHTSGVCAI
EU015061.1 SVTEHFNVYKATKPYLAYCADCGDGQFCYSPVAIEKIRDEASDGMIKIQVAAQIGINKGGTHEHNKIRYIAGHDMKEANRDSLQVHTSGVCAI
LC079086.1 SVTEHFNVYKATKPYLAYCADCGDGQFCYSPVAIEKIRDEASDGMIKIQVAAQIGINKGGTHEHNKIRYIAGHDMKEANRDSLQVHTSGVCAI
MN849355.1 SVTEHFNVYKATKPYLAYCADCGDGQSCYSPVAIEKIRDEASDGMIKIQVAAQIGINKGGTHEHNKIRYIAGHDMKEANRDSLQVHTSGTCAI
AB032553.1 SVTEHFNVYKATKPYLAYCADCGDGQFCYSPVAIEKIRDEASDGMIKIQVAAQIGINKRGTHEHNKIRYIVGHYMKEANRDSLQVHTSGVCAI
MK693225.1 SVTEHFNVYKATKPYLAYCADCGDGQFCYSPVAIEKIRDEASDGMIKIQVAAQIGINKGGTHEHNKIRYIAGHDMKEANRDSLQVHTSGVCAI
OK423758.1 SVTKHFNVYKATKPYLAYCADCGDGQFCYSPVAIEKIRDEASDGMIKIQVAAQIGINKGGTHEHNKIRYIAGHDMKEANRDSLQVHTSGVCAI
KY434327.1 SVTEHFNVYKATKPYLAYCADCGDGQFCYSPVAIEKIRDEASDGMIKIQVAAQIGINKGGTHEHNKIRYIAGHDMKEANRDSLQVYTSGVCAI
PQ846026.1 SVTEHFNVYKATKPYLAYCADCGDGQFCYSPVAIEKIRDEASDGMIKIQVAAQIGINKGGTHEHNKIRYIAGHDMKEANRDSLQVHTSGVCAI
PV235474.1 SVTEHFNVYKATKPYLAYCADCGDGQFCYSPVAIEKIRDEASDGMIKIQVAAQIGINKGGTHEHNKIRYIAGHDMKEANRDSLQVHTSGVCAI
100 110 120 130 140 150 160 170 180
OR487192.1 RGTMGHFIVAYCPPGDELKVQFQDAESHTQACKVQYKHAPAPVGREKFTVRPHFGIEVPCTTYQLTTAPTEEEIDMHTPPDIPDITLLSQQSG
AY702913.1 RGTMGHFIVAYCPPGDELKVQFQDAESHTQACKVQYKHAPAPVGREKFTVRPHFGIEVPCTTYQLTTAPTEEEIDMHTPPDIPDITLLSQQSG
PQ034602.1 RGTMGHFIVAYCPPGDELKVQFQDAESHTQACKVQYKHAPAPVGREKFTVRPHFGIEVPCTTYQLTTAPTEEEIDMHTPPDIPDITLLSQQSG
OR373097.1 RGTMGHFIVAYCPPGNELNVQFKDAESHTQACKVQYKHAPVPVGREKFTVRPHFGIEVPCTTYQLTTAPTEEEIDMHTPPDIPDITLLSQQSG
OP593308.1 RGTMGHFIVAYCPPGNELKVQFKDAESHTQACKVQYKHAPVPVGREKFTVRPHFGIEVPCTTYQLTTAPTEEEIDMHTPPDIPDITLLSQQSG
PQ662933.1 RGTMGHFIVAYCPPGDELKVQFQDAESHTQACKVQYKHAPAPVGREKFTVRPHFGIEVPCTTYQLTTAPTEEEIDMHTPPDIPDITLLSQQSG
LC152056.1 RGTMGHFIVAYCPPGDELKVQFQDAESHTQACKVQYKHAPAPVGREKFTVRPHFGIEVPCTTYQLTTAPTEEEIDMHTPPDIPDITLLSQQSG
LC079088.1 RGTMGHFIVAYCPPGDELKVQFQDAESHTQACKVQYKHAPAPVGREKFTVRPHFGIEVPCTTYQLTTAPTEEEIDMHTPPDIPDITLLSQQSG
LC212972.1 RGTMGHFIVAYCPPGDELKVQFQDAESHTQACKVQYKHAPAPVGREKFTVRPHFGIEVPCTTYQLTTAPTEEEIDMHTPPDIPDITLLSQQSG
LC223131.1 RGTMGHFIVAYCPPGDELKVQFQDAESHTQACKVQYKHAPAPVGREKFTVRPHFGIEVPCTTYQLTTAPTEEEIDMHTPPDIPDITLLSQQSG
EF011023.1 RGTMGHFIVAYCPPGGELKVQFQDAESHTQACKVQYKHAPAPVGREKFTVRPHFGIEVPCTTYQLTTAPTEEEIDMHTPPDIPDITLLSQQSG
MZ736788.1 RGTMGHFIVAYCPPGDELKVQFQDAESHTQACKVQYKHAPAPVGREKFTVRPHFGIEVPCTTYQLTTAPTEEEIDMHTPPDIPDITLLSQQSG
ON987235.1 RGTMGHFIVAYCPPGDELKVQFQDAESHTQACKVQYKHAPAPVGREKFTVRPHFGIEVPCTTYQLTTAPTEEEIDMHTPPDIPDITLLSQQSG
MT086508.1 RGTMGHFIVAYCPPGDELKVQFQDAESHTQACKVQYKHAPAPVGREKFTVRPHFGIEVPCTTYQLTTAPTEEKIDMHTPPDIPDITLLSQQSG
OQ863732.1 RGTMGHFIVAYCPPGDELKVQFQDAESHTQACKVQYKHAPAPVGREKFTVRPHFGIEVPCTTYQLTTAPTEEEIDMHTPPDIPDITLLSQQSG
MZ388464.1 RGTMGHFIVAYCPTGDELKVQFQDAESHTQACKVQYKHAPAPVGREKFTVRPHFGIEVPCTTYQLTTAPTEEEIDMHTPPDIPDITLLSQQSG
MZ357112.1 RGTMGHFIVAYCPPGDELKVQFQDAESHTQACKVQYKHAPAPVGREKFTVRPHFGIEVPCTTYQLTTAPTEEEIDMHTPPDIPDITLLSQQSG
MZ357111.1 RGTMGHFIVAYCPPGDELKVQFQDAESHTQACKVQYKHAPAPVGREKFTVRPHFGIEVPCTTYQLTTAPTEEEIDMHTPPDIPDITLLSQQSG
LC534253.1 RGTMGHFIVAYCPPGNELKVQFKDAESHTQACKVQYKHAPVPVGREKFTVRPHFGIEVPCTTYQLTTAPTEEEIDMHTPPDIPDITLLSQQSG
MZ736796.1 RGTMGHFIVAYCPPGDELKVQFQDAESHTQACKVQYKHAPAPVGREKFTVRPHFGIEVPCTTYQLTTAPTEEEIDMHTPPDIPDITLLSQQSG
MT269657.1 RGTMGHFIVAYCPPGDELKVQFQDAESHTQACKVQYKHAPAPVGREKFTVRPHFGIEVPCTTYQLTTAPTEEEIDMHTPPDIPDITLLSQQSG
MZ736801.1 RGTMGHFIVAYCPPGDELKVQFQDAESHTQACKVQYKHAPAPVGREKFTVRPHFGIEVPCTTYQLTTAPTEEEIDMHTPPDIPDITLLSQQSG
KY363862.1 RGTMGHFIVAYCPPGDELKVQFQDAESHTQACKVQYKHAPAPVGREKFTVRPHFGIEVPCTTYQLTTAPTEEEIDMHTPPDIPDITLLSQQSG
KY363863.1 RGTMGHFIVAYCPPGDELKVQFQDAESHTQACKVQYKHAPAPVGREKFTVRPHFGIEVPCTTYQLTTAPTEEEIDMHTPPDIPDITLLSQQSG
MG865966.1 RGTMGHFIVAYCPPGDELKVQFQDAESHTQACKVQYKHAPAPVGREKFTVRPHFGIEVPCTTYQLTTAPTEEEIDMHTPPDIPDITLLSQQSG
MF741771.1 RGTMGHFIVAYCPPGDELKVQFQDAESHTQACKVQYKHAPAPVGREKFTVRPHFGIEVPCTTYQLTTAPTEEEIDMHTPPDIPDITLLSQQSG
MG869691.1 RGTMGHFIVAYCPPGDELKVQFQDAESHTQACKVQYKHAPAPVGREKFTVRPHFGIEVPCTTYQLTTAPTEEEIDMHTPPDIPDITLLSQQSG
MH722256.1 RGTMGHFIVAYCPPGDELKVQFQDAESHTQACKVQYKHAPAPVGREKFTVRPHFGIEVPCTTYQLTTAPTEEEIDMHTPPDIPDITLLSQQSG
AB859822.1 RGTMGHFIVAYCPPGDELKVQFQDAESHTQACKVQYKHAPAPVGREKFTVRPHFGIEVPCTTYQLTTAPTEEEIDMHTPPDIPDITLLSQQSG
EF631998.1 RGTMGHFIVAYCPPGDELKVQFQDAESHTQACKVQYKHAPAPVGREKFTVRPHFGIEVPCTTYQLTTAPTEEEIDMHTPPDIPDITLLSQQSG
EF631999.1 RGTMGHFIVAYCPPGDELKVQFQDAESHTQACKVQYKHAPAPVGREKFTVRPHFGIEVPCTTYQLTTAPTEEEIDMHTPPDIPDITLLSQQSG
MW410934.1 RGTMGHFIVAYCPPGDELKVQFQDAESHTQACKVQYKHDPAPVGREKFTVRPHFGIEVPCTTYQLTTAPTEEEIDMHTPPDIPDITLLSQQSG
EU015061.1 RGTMGHFIVAYCPPGGELKVQFQDAESHTQACKVQYKHAPAPVGREKFTVRPHFGIEVPCTTYQLTTAPTEEEIDMHTPPDIPDITLLSQQSG
LC079086.1 RGTMGHFIVAYCPPGDELKVQFQDAESHTQACKVQYKHAPAPVGREKFTVRPHFGIEVPCTTYQLTTAPTEEEIDMHTPPDIPDITLLSQQSG
MN849355.1 RGTMGHFIAAYCPPGDELKVQFQDAESHIQACKVQYKHAPAPVGREKFTVRPHFGIEVPCTTYQLTTAPTEEEIDMHTPPDIPDITLLSQQSG
AB032553.1 RGTMGHFIVAYCPPGDELKVQFQDAESHTQACKVQYKHDPAPVGREKFTVRPHFGIEVPCTTYQLTTAPTEEEIDMHTPPDIPDITLLSQQSG
MK693225.1 RGTMGHFIVAYCPPGDELKVQFQDAESHTQACKVQYKHAPAPVGREKFTVRPHFGIEVPCTTYQLTTAPTEEEIDMHTPPDIPDITLLSQQSG
OK423758.1 RGTMGHFIVAYCPPGDELKVQFQDAESHTQACKVQYKHAPAPVGREKFTVRPHFGIEVPCTTYQLTTAPTEEEIDMHTPPDIPDITLLSQQSG
KY434327.1 RGTMGHFIVAYCPPGNELKVQFKDAESHTQACKVQYKHAPVPVGREKFTVRPHFGIEVPCTTYQLTTAPTEEEIDMHTPPDIPDITLLSQQSG
PQ846026.1 RGTMGHFIVAYCPPGDELKVQFQDAESHTQACKVQYKHAPAPVGREKFTVRPHFGIEVPCTTYQLTTAPTEEEIDMHTPPDIPDITLLSQQSG
PV235474.1 RGTMGHFIVAYCPPGDELKVQFQDAESHTQACKVQYKHAPAPVGREKFTVRPHFGIEVPCTTYQLTTAPTEEEIDMHTPPDIPDITLLSQQSG

```
              190       200       210       220       230       240       250       260       270
OR487192.1  NVKITAGGKTIRYNCTCGSGNVGTTSSDKTINSCKIAQCHAAVTNHDKWQYTSSFVPRADQLSRKGKVHVPFPLTNSTCRVPVARAPGVTYGK
AY702913.1  NVKITAGGKTIRYNCTCGSGNVGTTSSDKTINSCKIAQCHAAVTNHDKWQYTSSFVPRADQLSRKGKVHVPFPLTNSTCRVPVARAPGVTYGK
PQ034602.1  NVKITAGGKTIRYNCTCGSGNVGTTSSDKTINSCKIAQCHAAVTNHDKWQYTSSFVPRADQLSRKGKVHVPFPLTNSTCRVPVARAPGVTYGK
OR373097.1  NVKITAGGKTIRYNCTCGNGHVGTTSTDKTINSCKIAQCHAAVTNHDKWQYTSSFVPRADQLSRKGKVHVPFPLTNSTCRVPVARAPGVTYGK
OP593308.1  NVKITAGGKTIRYNCTCGNGNVGTTSTDKTINSCKIAQCHAAVTNHDKWQYTSSFVPRADRLSRKGKVHVPFPLTNSTCRVPVARAPGVTYGK
PQ662933.1  NVKITAGGKTIRYNCTCGSGNVGTTSSDKTINSCKIAQCHAAVTNHDKWQYTSSFVPRADQLSRKGKVHVPFPLTNSTCRVPVARAPGVTYGK
LC152056.1  NVKITAGGKTIRYNCTCGSGNVGTTSSDKTINSCKIAQCHAAVTNHDKWQYTSSFVPRADQLSRKGKVHVPFPLTNSTCRVPVARAPGVTYGK
LC079088.1  NVKITAGGKTIRYNCTCGSGNVGTTSSDKTINSCKIAQCHAAVTNHDKWQYTSSFVPRADQLSRKGKVHVPFPLTNSTCRVPVARAPGVTYGK
LC212972.1  NVKITAGGKTIRYNCTCGSGNVGTTSSDKTINSCKIAQCHAAVTNHDKWQYTSSFVPRADQLSRKGKVHVPFPLTNSTCRVPVARAPGVTYGK
LC223131.1  NVKITAGGKTIRYNCTCGSGNVGTTSSDKTINSCKIAQCHAAVTNHDKWQYTSSFVPRADQLSRKGKVHVPFPLTNSTCRVPVARAPGVTYGK
EF011023.1  NVKITAGGKTIRYNCTCGSGNVGTTSSDKTINSCKIAQCHAAVTNHDKWQYTSSFVPRADQLSRKGKVHVPFPLTNSTCRVPLARAPGVTYGK
MZ736788.1  NVKITAGGKTIRYNCTCGSGNVGTTSSDKTINSCKIAQCHAAVTNHDKWQYTSSFVPRADQLSRKGRVHVPFPLTNSTCRVPVARAPGVTYGK
ON987235.1  NVKITAGGKTIRYNCTCGSGNVGTTSSDKTINSCKIAQCHAAVTNHDKWQYTSSFVPRADQLSRKGKVHVPFPLTNSTCRVPVARAPGVTYGK
MT086508.1  NVKITAGGKTIRYNCTCGSGNVGTTSSDKTINSCKIAQCHAAVTNHDKWQYTSSFVPRADQLSRKGKVHVPFPLTNSTCRVPVARAPGVTYGK
OQ863732.1  NVKITAGGKTIRYNCTCGSGNVGTTSSDKTINSCKIAQCHAAVTNHDKWQYTSSFVPRADQLSRKGKVHVPFPLTNSTCRVPVARAPGVTYGK
MZ388464.1  NVKITAGGKTIRYNCTCGSGNVGTTSSDKTINSCKIAQCHAAVTNHDKWQYTSSFVPRADQLSRKGKVHVPFPLTNSTCRVPVARAPGVTYGK
MZ357112.1  NVKITAGGKTIRYNCTCGSGNVGTTSSDKTINSCKIAQCHAAVTNHDKWQYTSSFVPRADQLSRKGKVHVPFPLTNSTCRVPVARAPGVTYGK
MZ357111.1  NVKITAGGKTIRYNCTCGSGNVGTTSSDKTINSCKIAQCHAAVTNHDKWQYTSSFVPRADQLSRKGKVHVPFPLTNSTCRVPVARAPGVTYGK
LC534253.1  NVKITAGGKTIRYNCTCGNGNVGTTSTDKTINSCKIAQCHAAVTNHDKWQYTSSFVPRADQLSRKGKVHVPFPLTNSTCRVPVARAPGVTYGK
MZ736796.1  NVKITAGGKTIRYNCTCGSGNVGTTSSDKTINSCKIAQCHAAVTNHDKWQYTSSFVPRADQLSRKGKVHVPFPLTNSTCRVPVARAPGVTYGK
MT269657.1  NVKITAGGKTIRYNCTCGSGHVGTTSSDKTINSCKIAQCHAAVTNHDKWQYTSSFVPRADQLSRKGKVHVPFPLTNSTCRVPVARTPGVTYGK
MZ736801.1  NVKITAGGKTIRYNCTCGSGNVGTTSSDKTINSCKIAQCHAAVTNHDKWQYTSSFVPRADQLSRKGKVHVPFPLTNSTCRVPVARAPGVTYGK
KY363862.1  NVKITAGGKTIRYNCTCGSGNVGTTSSDKTINSCKIAQCHAAVTNHDKWQYTSSFVPRADQLSRKGKVHVPFPLTNSTCRVPVARAPGVTYGK
KY363863.1  NVKITAGGKTIRYNCTCGSGNVGTTSSDKTINSCKIAQCHAAVTNHDKWQYTSSFVPRADQLSRKGKVHVPFPLTNSTCRVPVARAPGVTYGK
MG865966.1  NVKITAGGKTIRYNCTCGSGNVGTTSSDKTINSCKIAQCHAAVTNHDKWQYTSSFVPRADQLSRKGKVHVPFPLTNSTCRVPVARAPGVTYGK
MF741771.1  NVKITAGGKTIRYNCTCGSGHVGTTSSDKTINSCKIAQCHAAVTNHDKWQYTSSFVPRADQLSRKGKVHVPFPLTNSTCRVPVARAPGVTYGK
MG869691.1  NVKITAGGKTIRYNCTCGSGNVGTTSSDKTINSCKIAQCHAAVTNHDKWQYTSSFVPRADQLSRKGKVHVPFPLTNSTCRVPVARAPGVTYGK
MH722256.1  NVKITAGGKTIRYNCTCGSGHVGTTSSDKTINSCKIAQCHAAVTNHDKWQYTSSFVPRADQLSRKGKVHVPFPLTNSTCRVPVARAPGVTYGK
AB859822.1  NVKITAGGKTIRYNCTCGSGHVGTTSSDKTINSCKIAQCHAAVTNHDKWQYTSSFVPRADQLSRKGKVHVPFPLTNSTCRVPVARAPGVTYGK
EF631998.1  NVKITAGGKTIRYNCTCGSGNVGTTSSDKTINSCKIAQCHAAVTNHDKWQYTSSFVPRADQLSRKGKVHVPFPLTNSTCRVPLARAPGVTYGK
EF631999.1  NVKITAGGKTIRYNCTCGSGNVGTTSSDKTINSCKIAQCHAAVTNHDKWQYTSSFVPRADQLSRKGKVHVPFPLTNSTCRVPVARAPGVTYGK
MW410934.1  NVKITAGGKTIRYNCTCGSGNVGTTSSDKTINSCKIAQCHAAVTNHDKWQYTSSFVPRADQLPRKGKVHVPFPLTNSTCRVPLARAPGVTYGK
EU015061.1  NVKITAGGKTIRYNCTCGRGNVGTTSSDKTINSCKIAQCHAAVTNHDKWQYTSSFVPRADQLSRKGKVHVPFPLTNSTCRVPLARAPGVTYGK
LC079086.1  NVKITAGEKTIRYNCTCGSGNVGTTSSDKTINSCKIAQCHAAVTNHDKWQYTSSFVPRADQLSRKGKVHVPFPLTNSTCRVPVARAPGVTYGK
MN849355.1  NVKITAGGKTIRYNCTCGSGNVGTTSSDKTINSCKIAQCHAAVTNHDKWQYTSSFVPRADQLSRKGKVHVPFPLTDSTCRVPLARAPGVTYGK
AB032553.1  DVKITAGGKTIRYNCTCGSGNVGTTSSDKTINSCKIAQCHAAVTNHDKWQYTSSFVPRADQLPRKGKVHVPFPLTNSTCRVPLARAPGVTYGK
MK693225.1  NVKITAGGKTIRYNCTCGSGNVGTTSSDKTINSCKIAQCHAAVTNHDKWQYTSSFVPRADQLSRKGKVHVPFPLTNSTCRVPVARAPGVTYGK
OK423758.1  NVKITAGGKTIRYNCTCGSGNVGTTSSDKTINSCKIAQCHAAVTNHDKWQYTSSFVPRADQLSRKGKVHVPFPLTNSTCRVPVARAPGVTYGK
KY434327.1  NVKITAGGKTIRYNCTC.NGNVGTTSSDKTINSCKIAQCHAAVTNHDKWQYTSSFVPRADQLSRKGKVHVPFPLTNSTCRVPLARAPGVTYGK
PQ846026.1  NVKITAGGKTIRYNCTCGSGNVGTTSSDKTINSCKIAQCHAAVTNHDKWQYTSSFVPRADQLSRKGKVHVPFPLTNSTCRVPVARAPGVTYGK
PV235474.1  NVKITAGGKTIRYNCTCGSGNVGTTSSDKTINSCKITQCHAAVTNHDKWQYTSSFVPRADQLSRKGKVHVPFPLTNSTCRVPVARAPGVTYGK
```

```
            280       290       300       310       320       330       340       350       360       370
OR487192.1  RELTVKLHPDHPTLLTYRSLGADPRPYEEWIDRYVERTIPVTEEGIEYRWGNNPPVRLWAQLTTEGKPHGWPHEIILYYYGLYPAATIAAVSA
AY702913.1  RELTVKLHPDHPTLLTYRSLGADPRPYEEWIDRYVERTIPVTEDGIEYRWGNNPPVRLWAQLTTEGKPHGWPHEIILYYYGLYPAATIAAVSA
PQ034602.1  RELTVKLHPDHPTLLTYRSLGADPRPYEEWIDRYVERTIPVTEEGIEYRWGNNPPVRLWAQLTTEGKPHGWPHEIILYYYGLYPAATIAAVSA
OR373097.1  RELTVKLHPDHPTLLTYRSLGADPRPHEEWIDQYVERTIPVTEDGIEYRWGNNPPVRLWAQLTTEGKPHGWPHEIILYYYGLYPAATIAAVSA
OP593308.1  RELTVKLHPDHPTLLTYRSLGADPRPHEEWIDQYVERTIPVTEDGIEYRWGNNPPVRLWAQLTTEGKPHGWPHEIILYYYGLYPAATIAAVSA
PQ662933.1  RELTVKLHPDHPTLLTYRSLGADPRPYEEWIDRYVERTIPVTEDGIEYRWGNNPPVRLWAQLTTEGKPHGWPHEIILYYYGLYPAATVAAVSA
LC152056.1  RELTVKLHPDHPTLLTYRSLGADPRPYEEWIDRYVERTIPVTEDGIEYRWGNNPPVRLWAQLTTEGKPHGWPHEIILYYYGLYPAATIAAVSA
LC079088.1  RELTVKLHPDHPTLLTYRSLGADPRPYEEWIDRYVERTIPVTEDGIEYRWGNNPPVRLWAQLTTEGKPHGWPHEIILYYYGLYPAATIAAVSA
LC212972.1  RELTVKLHPDHPTLLTYRSLGADPRPYEEWIDRYVERTIPVTEDGIEYRWGNNPPVRLWAQLTTEGKPHGWPHEIILYYYGLYPAATIAAVSA
LC223131.1  RELTVKLHPDHPTLLTYRSLGADPRPYEEWIDRYVERTIPVTEDGIEYRWGNNPPVRLWAQLTTEGKPHGWPHEIILYYYGLYPAATIAAVSA
EF011023.1  RELTVKLHPDHPTLLTYRSLGADPRPYEEWIDRYVERTIPVTEDGIEYRWGNNPPVRLWAQLTTEGKPHGWPHEIILYYYGLYPAATIVAVSA
MZ736788.1  RELTVKLHPDHPTLLTYRSLGADPRPYEEWIDRYVERTIPVTEDGIEYRWGNNPPVRLWAQLTTEGKPHGWPHEIILYYYGIYPAATIPTVST
ON987235.1  RELTVKLHPDHPTLLTYRSLGADPRPYEEWIDRYVERTIPVTEDGIEYRWGNNPPVRLWAQLTTEGKPHGWPHEIILYYYGLYPAATIAAVSA
MT086508.1  RELTVKLHPDHPTLLTYRSLGADPRPYEEWIDRYVERTIPVTEDGIEYRWGNNPPVRLWAQLTTEGKPHGWPHEIILYYYGLYPAATIAAVSA
OQ863732.1  RELTVKLHPDHPTLLTYRSLGADPRPYEEWIDRYVERTIPVTEDGIEYRWGNNPPVRLWAQLTTEGKPHGWPHEIILYYYGLYPAATIAAVSA
MZ388464.1  RELTVKLHPDHPTLLTYRSLGADPRPYEEWIDRYVERTIPVTEEGIEYRWGNNPPVRLWAQLTTEGKPHGWPHEIILYYYGLYPAATIAAVSA
MZ357112.1  RELTVKLHPDHPTLLTYRSLGADPRPYEEWIDRYVERTIPVTEEGIEYRWGNNPPVRLWAQLTTEGKPHGWPHEIILYYYGLYPAATIAAVSA
MZ357111.1  RELTVKLHPDHPTLLTYRSLGADPRPYEEWIDRYVERTIPVTEEGIEYRWGNNPPVRLWAQLTTEGKPHGWPHEIILYYYGLYPAATIAAVSA
LC534253.1  RELTVKLHPDHPTLLTYRSLGADPRPHEEWIDQYVERTIPVTEDGIEYRWGNNPPVRLWAQLTTEGKPHGWPHEIILYYYGLYPAATIAAVSA
MZ736796.1  RELTVKLHPDHPTLLTYRSLGADPRPYEEWIDRYVERTIPVTEEGIEYRWGNNPPVRLWAQLTTEGKPHGWPHEIILYYYGLYPAATIAAVSA
MT269657.1  RELTVKLHPDHPTLLTYRSLGADPRPYEEWIDRYVERTIPVTEDGIEYRWGNNPPVRLWAQLTTEGKPHGWPHEIILYYYGLYPAATIAAVSA
MZ736801.1  RELTVKLHPDHPTLLTYRSLGADPRPYEEWIDRYVERTIPVTEDGIEYRWGNNPPVRLWAQLTTEGKPHGWPHEIILYYYGLYPAATIAAVSA
KY363862.1  RELTVKLHPDHPTLLTYRSLGADPRPYEEWIDRYVERTIPVTEDGIEYRWGNNPPVRLWAQLTTEGKPHGWPHEIILYYYGLYPAATIAAVSA
KY363863.1  RELTVKLHPDHPTLLTYRSLGADPRPYEEWIDRYVERTIPVTEDGIEYRWGNNPPVRLWAQLTTEGKPHGWPHEIILYYYGLYPAATIAAVSA
MG865966.1  RELTVKLHPDHPTLLTYRSLGADPRPYEEWIDRYVERTIPVTEDGIEYRWGNNPPVRLWAQLTTEGKPHGWPHEIILYYYGLYPAATIAAVSA
MF741771.1  RELTVKLHPDHPTLLTYRSLGADPRPYEEWIDRYVERTIPVTEDGIEYRWGNNPPVRLWAQLTTEGKPHGWPHEIILYYYGLYPAATIAAVSA
MG869691.1  RELTVKLHPDHPTLLTYRSLGADPRPYEEWIDRYVERTIPVTEDGIEYRWGNNPPVRLWAQLTTEGKPHGWPHEIILYYYGLYPAATIAAVSA
MH722256.1  RELTVKLHPDHPTLLTYRSLGADPRPYEEWIDRYVERTIPVTEDGIEYRWGNNPPVRLWAQLTTEGKPHGWPHEIILYYYGLYPAATIAAVSA
AB859822.1  RELTVKLHPDHPTLLTYRSLGADPRPYEEWIDRYVERTIPVTEDGIEYRWGNNPPVRLWAQLTTEGKPHGWPHEIILYYYGLYPAATIAAVSA
EF631998.1  RELTVKLHPDHPTLLTYRSLGADPRPYEEWIDRYVERTIPVTEDGIEYRWGNNPPVRLWAQLTTEGKPHGWPHEIILYYYGLYPAATIAAVSA
EF631999.1  RELTVKLHPDHPTLLTYRSLGADPRPYEEWIDRYVERTIPVTEDGIEYRWGNNPPVRLWAQLTTEGKPHGWPHEIILYYYGLYPAATIAAVSA
MW410934.1  RELTVKLHPDHPTLLTYRSLGADPRPYEEWIDRYVERTIPVTEDGIEYRWGNNPPVRLWAQLTTEGKPHGWPHEIILYYYGLYPAATIAAVSA
EU015061.1  RELTVKLHPDHPTLLTYRSLGADPRPYEEWIDRYVERTIPVTEDGIEYRWGNNPPVRLWAQLTTEGKPHGWPHEIILYYYGLYPAATIVAVSA
LC079086.1  RELTVKLHPDHPTLLTYRSLGADPRPYEEWIDRYVERTIPVTEDGIEYRWGNNPPVRLWAQLTTEGKPHGWPHEIILYYYGLYPAATIAAVSA
MN849355.1  RELTVKLHPDHPTLLTYRSLGADPRPYEEWIDRYAERTIPVTEEGIEYRWGNNPPVRLWAQLTTEGKPHGWPHEIILYYYGLYPAATIAAVSA
AB032553.1  RELTVKLHPDHPTLLTYRSLGADPRPYEEWIDRYVERTIPVTEDGIEYRWGNNPPVRLWAQLTTEGKPHGWPHEIILYYYGLYPAATIAAVSA
MK693225.1  RELTVKLHPDHPTLLTYRSLGADPRPYEEWIDRYVERTIPVTEEGIEYRWGNNPPVRLWAQLTTEGKPHGWPHEIILYYYGLYPAATIAAVSA
OK423758.1  RELTVKLHPDHPTLLTYRSLGADPRPYEEWIDRYVERTIPVTEEGIEYRWGNNPPVRLWAQLTTEGKPHGWPHEIILYYYGLYPAATIAAVSA
KY434327.1  RELTVKLHPDHPTLLTYRSLGADPRPYEEWIDQYVERTIPVTEDGIEYRWGNNPPVRLWAQLTTEGKPHGWPHEIILYYYGLYPAATIAAVSA
PQ846026.1  RELTVKLHPDHPTLLTYRSLGADPRPYEEWIDRHVERTIPVTEDGIEYRWGNNPPVRLWAQLTTEGKPHGWPHEIILYYYGLYPAATIAAVSA
PV235474.1  RELTVKLHPDHPTLLTYRSLGADPRPYEEWIDRHVERTIPVTEDGIEYRWGNNPPVRLWAQLTTEGKPHGWPHEIILYYYGLYPAATIAAVSA
```

```
                   380       390       400       410       420
OR487192.1   VGLAVVLSLLASCYMFATARRKCLTPYALTPGAVVPVTLGVLCCAPRAHA
AY702913.1   AGLAVVLSLLASCYMFATARRKCLTPYALTPGAVVPVTLGVLCCAPRAHA
PQ034602.1   AGLAVVLSLLASCYMFATARRKCLTPYALTPGAVVPVTLGVLCCAPRAHA
OR373097.1   AGLTVVLSLLASCYMFATARRKCLTPYALTPGAVVPVTLGVLCCAPRAHA
OP593308.1   AGLTVVLSLLASCYMFATARRKCLTPYALTPGAVVPVTLGVLCCAPRAHA
PQ662933.1   AGLAVVLSLLASCYMFATARRKCLTPYALTPGAVVPVTLGVLCCAPRAHA
LC152056.1   AGLAVVLSLLASCYMFATARRKCLTPYALTPGAVVPVTLGVLCCAPRAHA
LC079088.1   AGLAVVLSLLASCYMFATARRKCLTPYALTPGAVVPVTLGVLCCAPRAHA
LC212972.1   AGLAVVLSLLASCYMFATARRKCLTPYALTPGAVVPVTLGVLCCAPRAHA
LC223131.1   AGLAVVLSLLASCYMFATARRKCLTPYALTPGAVVPVTLGVLCCAPRAHA
EF011023.1   ACLAVVLSLLASCYMFATARRKCLTPYALTPGAVVPVTLGVLCCAPRAHA
MZ736788.1   AGLAVVLSLLASCYMFATARRKCLTPYALTPGAVVPVTLGVLCCAPRAHA
ON987235.1   AGLAVVLSLLASCYMFATARRKCLTPYALTPGAVVPVTLGVLCCAPRAHA
MT086508.1   AGLAVVLSLLASCYMFATARRKCLTPYALTPGAVVPVTLGVLCCAPRAHA
OQ863732.1   AGLAVVLSLLASCYMFATARRKCLTPYALTPGAVVPVTLGVLCCAPRAHA
MZ388464.1   AGLAVVLSLLASCYMFATARRKCLTPYALTPGAVVPVTLGVLCCAPRAHA
MZ357112.1   AGLAVVLSLLASCYMFATARRKCLTPYALTPGAVVPVTLGVLCCAPRAHA
MZ357111.1   AGLAVVLSLLASCYMFATARRKCLTPYALTPGAVVPVTLGVLCCAPRAHA
LC534253.1   AGLTVVLSLLASCYMFATARRKCLTPYALTPGAVVPVTLGVLCCAPRAHA
MZ736796.1   AGLAVVLSLLASCYMFATARRKCLTPYALTPGAVVPVTLGVLCCAPRAHA
MT269657.1   AGLAAILSLLASCYMFATARRKCLTPYALTPGAVIPVTLGVLCCAPRAHA
MZ736801.1   AGLAVVLSLLASCYMFATARRKCLTPYALTPGAVVPVTLGVLCCAPRAHA
KY363862.1   AGLAVVLSLLASCYMFATARRKCLTPYALTPGAVVPVTLGVLCCAPRAHA
KY363863.1   AGLAVVLSLLASCYMFATARRKCLTPYALTPGAVVPVTLGVLCCAPRAHA
MG865966.1   AGLAVVLSLLASCYMFATARRKCLTPYALTPGAVVPVTLGVLCCAPRAHA
MF741771.1   AGLAAILSLLASCYMFATARRKCLTPYALTPGAVIPVTLGVLCCAPRAHA
MG869691.1   AGLAVVLSLLASCYMFATARRKCLTPYALTPGAVVPVTLGVLCCAPRAHA
MH722256.1   AGLAAILSLLASCYMFATARRKCLTPYALTPGAVIPVTLGVLCCAPRAHA
AB859822.1   AGLAVILSLLTSCYMFATARRKCLTPYALTPGAVIPVTLGVLCCAPRAHA
EF631998.1   AGLAVVLSLLASCYMFATARRKCLTPYALTPGAVVPVTLGVLCCAPRAHA
EF631999.1   AGLAVVLSLLASCYMFATARRKCLTPYALTPGAVVPVTLGVLCCAPRAHA
MW410934.1   AGLAVVLSLLASCYMFATARRKCLTPYALTPGAVVPVTLGVLCCAPRAHA
EU015061.1   ACLAVVLSLLASCYMFATARRKCLTPYALTPGAVVPVTLGVLCCAPRAHA
LC079086.1   AGLAVVLSLLASCYMFATARRKCLTPYALTPGAVVPVTLGVLCCAPRAHA
MN849355.1   AGLAVVLSLLASCYMFATARRKCLTPYALTPGAIVPVTLGVLCCAPRAHA
AB032553.1   AGLAVVLSLLASCYMFATARRKCLTPYALTPGAVVPVTLGVLCCAPRAHA
MK693225.1   AGLAVVLSLLASCYMFATARRKCLTPYALTPGAVVPVTLGVLCCAPRAHA
OK423758.1   VGLAVVLSLLASCYMFATARRKCLTPYALTPGAVVPVTLGVLCCAPRAHA
KY434327.1   AGLAVVLSLLASCYMFATARRKCLTPYALTPGAVVPVTLGVLCCAPRAHA
PQ846026.1   AGLAVVLSLLASCYMFATARRKCLTPYALTPGAVVPVTLGVLCCAPRAHA
PV235474.1   AGLAVVLSLLASCYMFATARRKCLTPYALTPGAVVPVTLGVLCCAPRAHA
```
