# Supplementary figures and images for: Re-Emergence and Characterization of a Highly Pathogenic Getah Virus on a Pig Farm in Guangdong Province, China
Source: Microorganisms. 2026 Apr 9;14(4):846. doi: 10.3390/microorganisms14040846 (PMC13119205; doi:10.3390/microorganisms14040846)

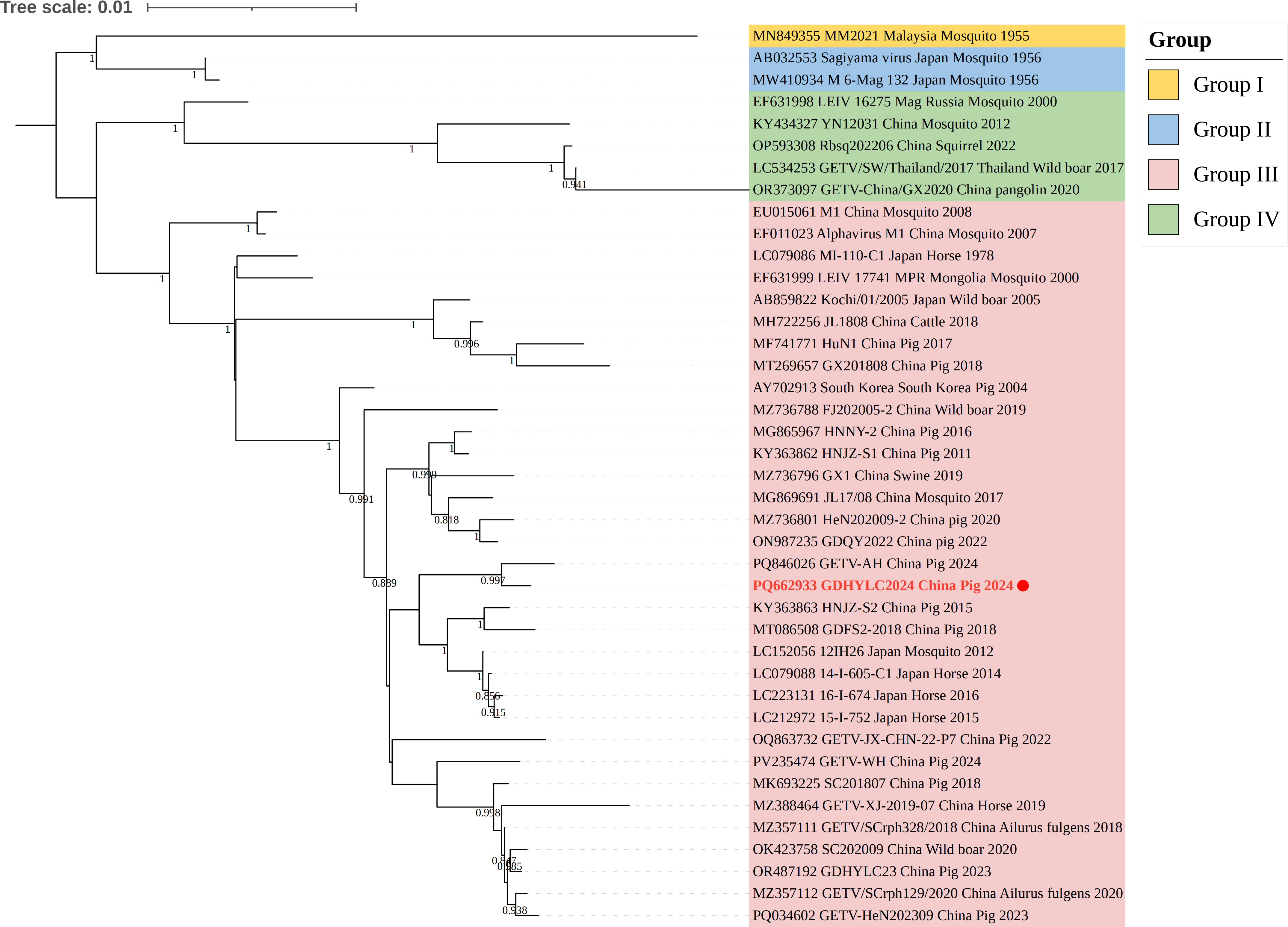

Supplement: Supplementary file 1 [file microorganisms-14-00846-s001.zip › microorganisms-4221267-supplementary/Supplementary_Figures.zip/Figures S2. Phylogenetic trees based on the non-structural gene.png]

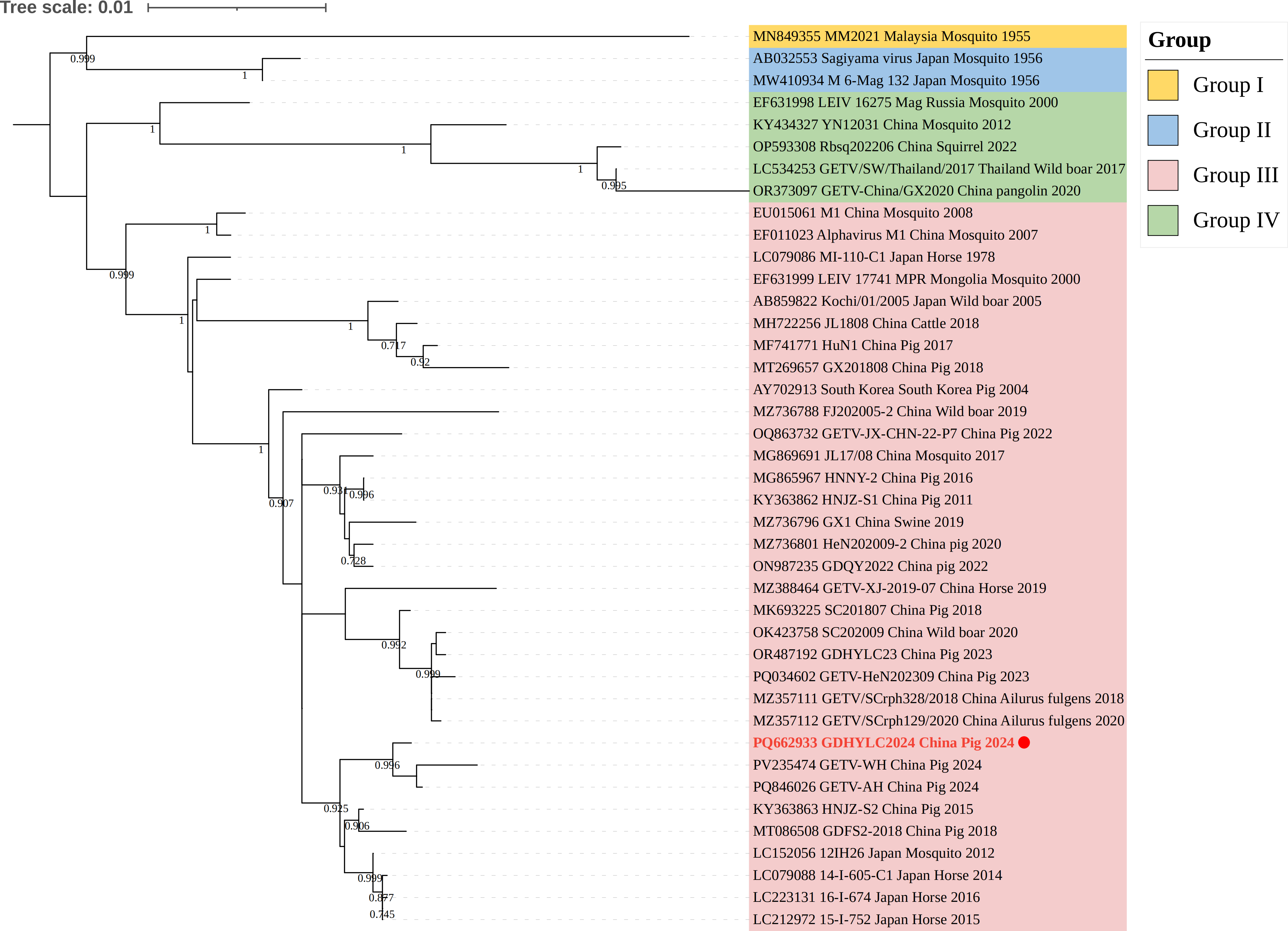

Supplement: Supplementary file 1 [file microorganisms-14-00846-s001.zip › microorganisms-4221267-supplementary/Supplementary_Figures.zip/Figures S3. Phylogenetic trees based on the structural gene.png]

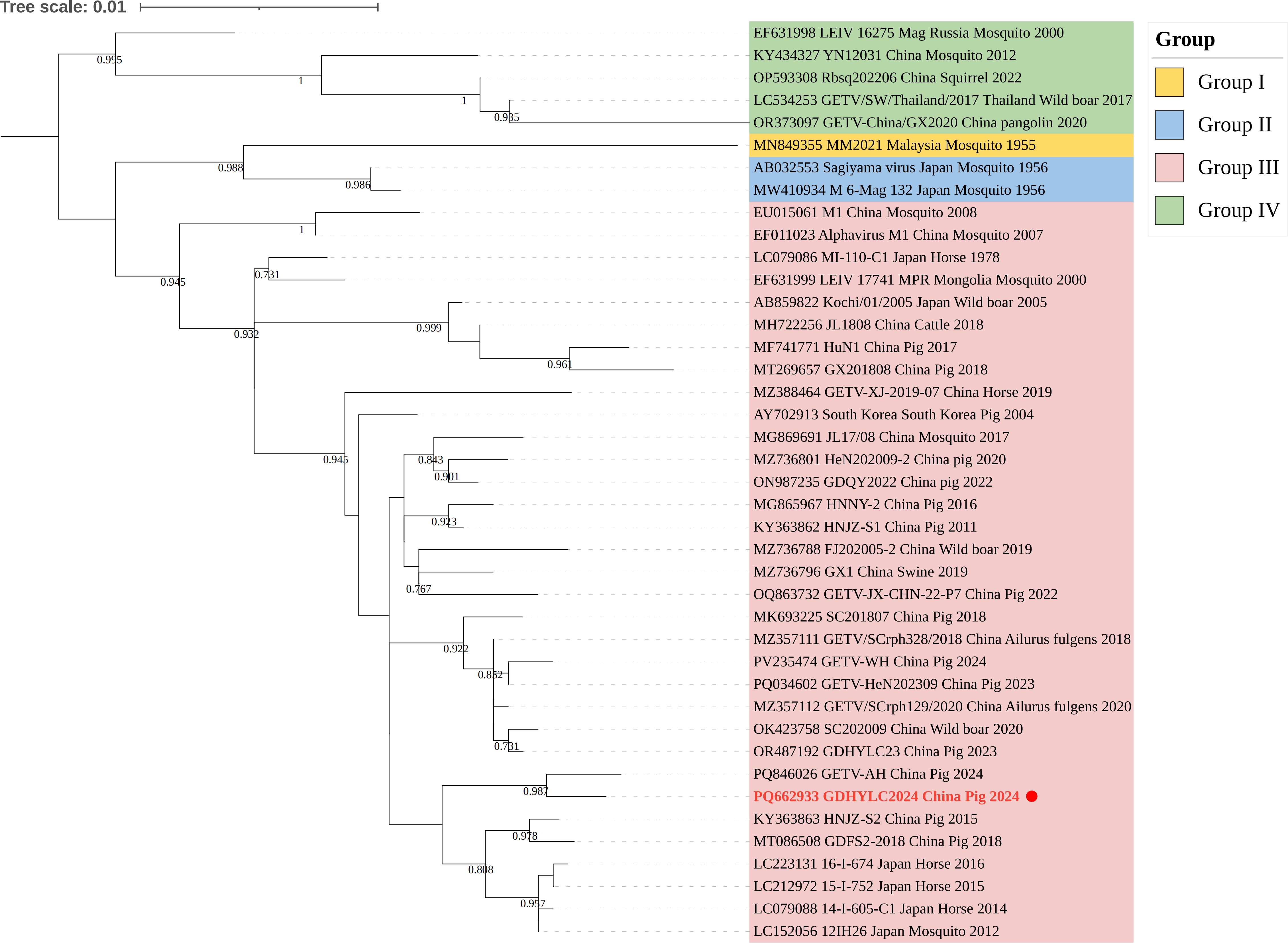

Supplement: Supplementary file 1 [file microorganisms-14-00846-s001.zip › microorganisms-4221267-supplementary/Supplementary_Figures.zip/Figures S4. Phylogenetic trees based on the NSP1 gene.png]

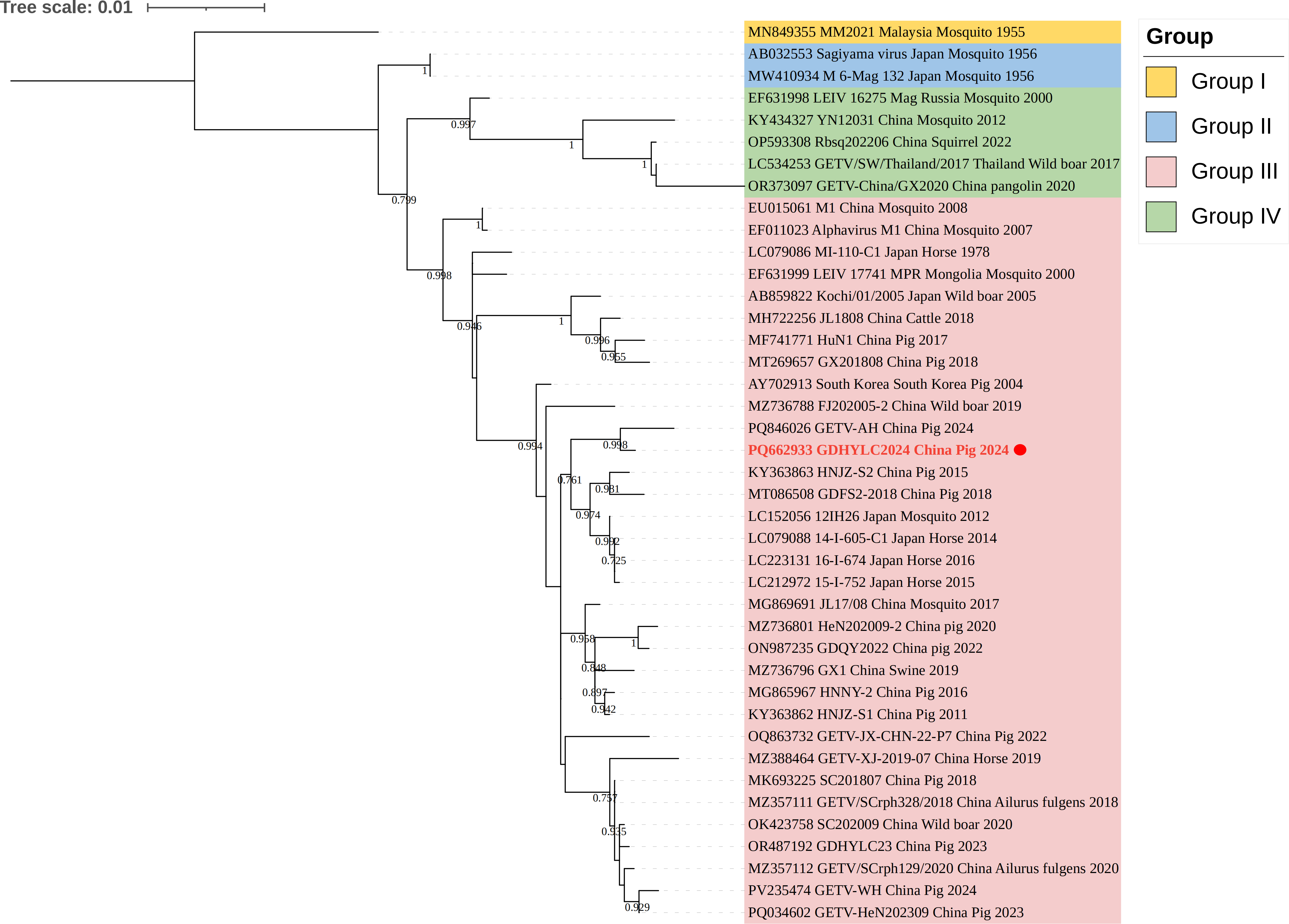

Supplement: Supplementary file 1 [file microorganisms-14-00846-s001.zip › microorganisms-4221267-supplementary/Supplementary_Figures.zip/Figures S5. Phylogenetic trees based on the NSP2 gene.png]

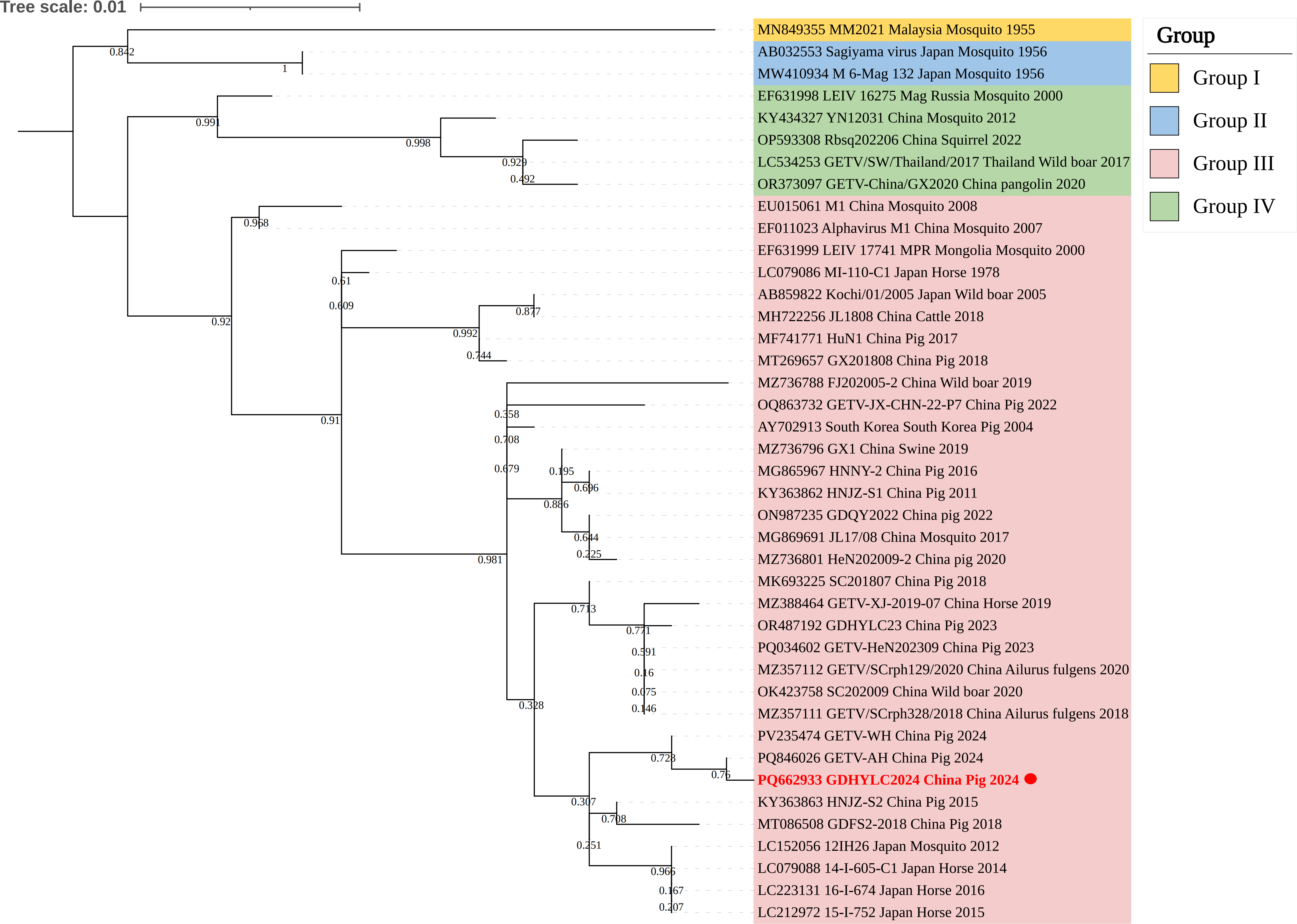

Supplement: Supplementary file 1 [file microorganisms-14-00846-s001.zip › microorganisms-4221267-supplementary/Supplementary_Figures.zip/Figures S6. Phylogenetic trees based on the Cap gene.png]

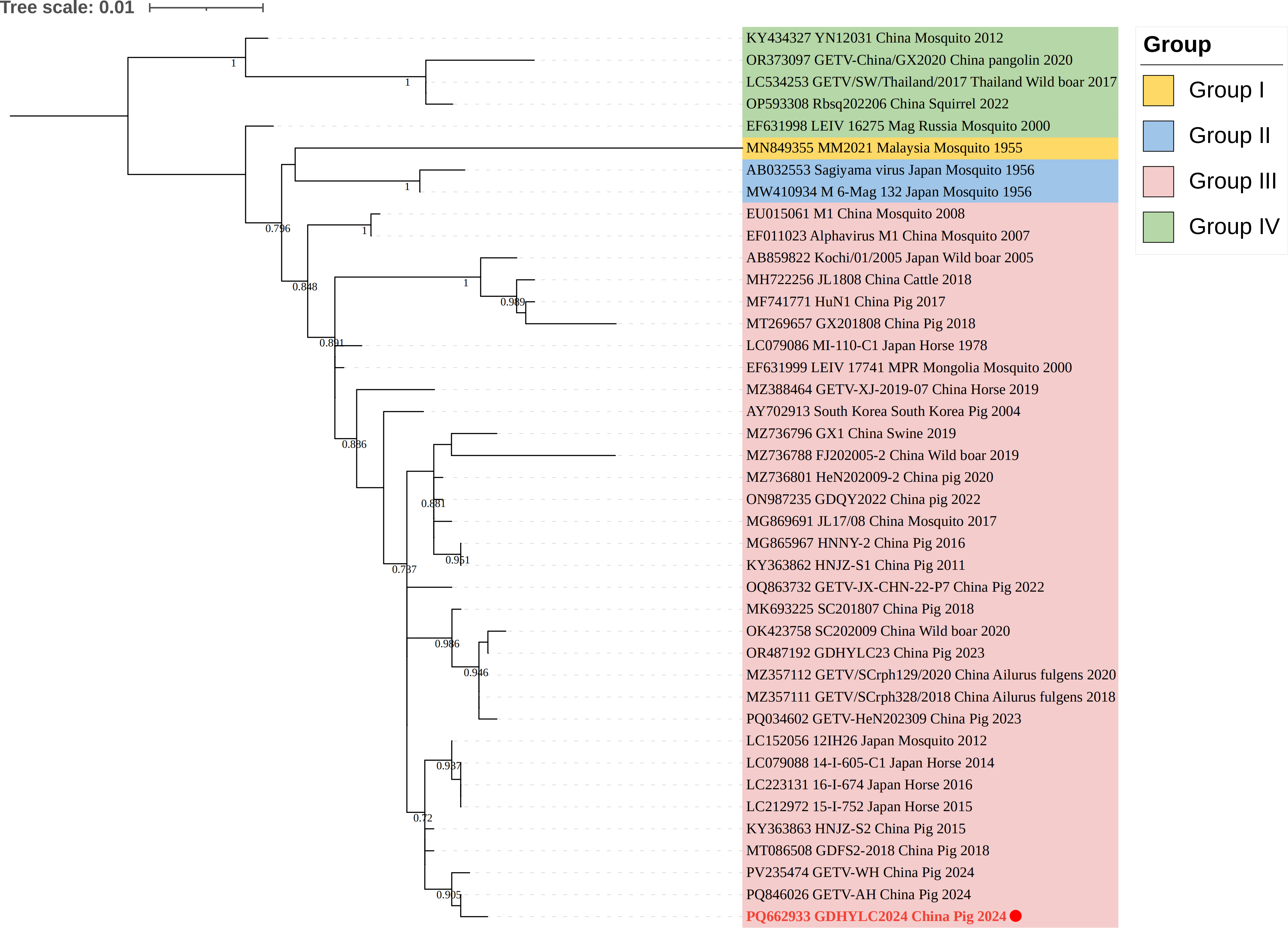

Supplement: Supplementary file 1 [file microorganisms-14-00846-s001.zip › microorganisms-4221267-supplementary/Supplementary_Figures.zip/Figures S7. Phylogenetic trees based on the E2 gene.png]

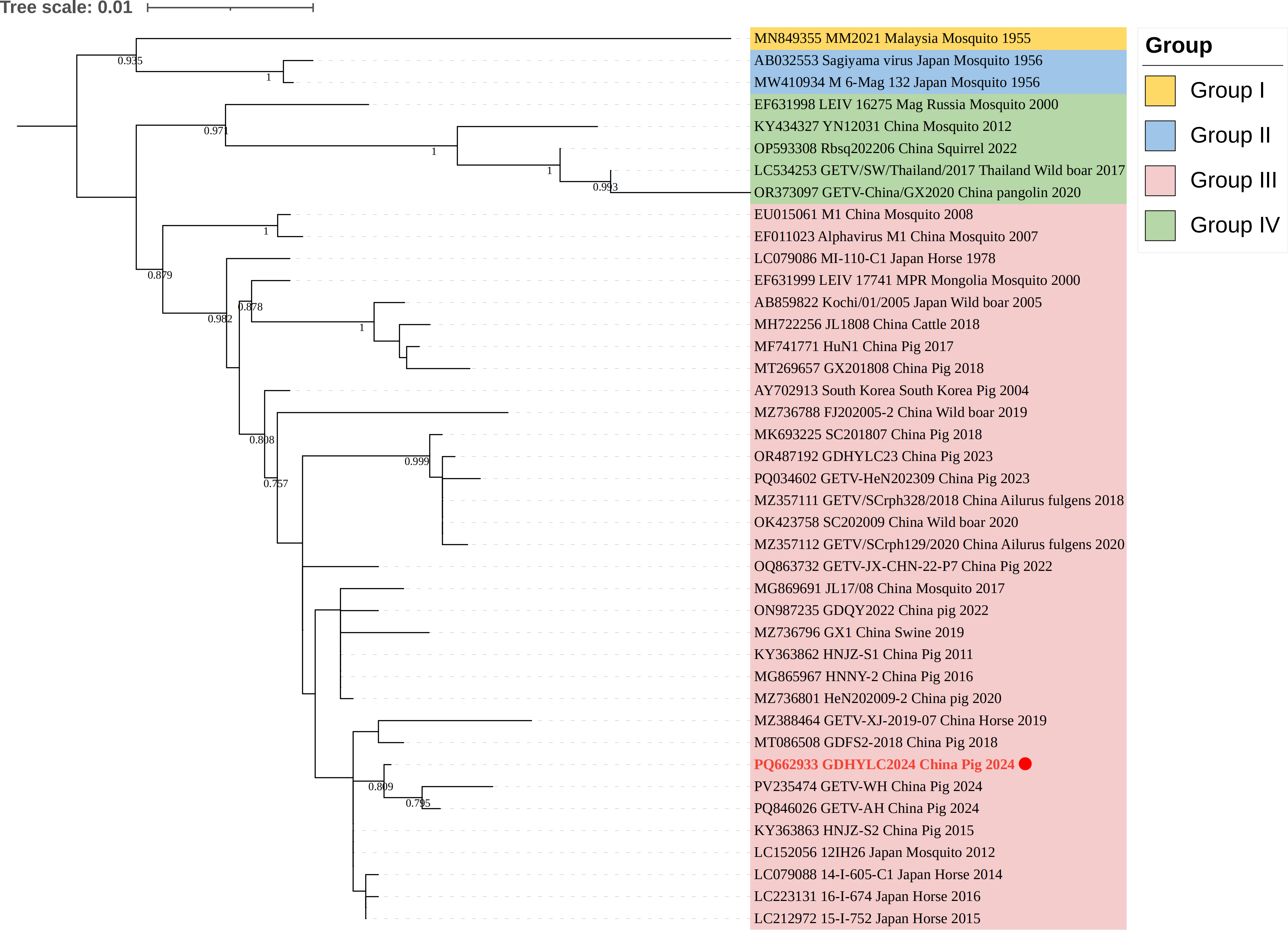

Supplement: Supplementary file 1 [file microorganisms-14-00846-s001.zip › microorganisms-4221267-supplementary/Supplementary_Figures.zip/Figures S8. Phylogenetic trees based on the E1 gene.png]
